# Supplementary material for: Complete atomic structure of a native archaeal cell surface
Source: Cell Rep. 2021 Nov 23;37(8):110052. doi: 10.1016/j.celrep.2021.110052 (PMC8640222; doi:10.1016/j.celrep.2021.110052)
Supplement: Document S1. Figures S1–S8 and Tables S1–S3 [file mmc1.pdf]

Cell Reports, Volume *37*

Supplemental information

Complete atomic structure of a native archaeal cell surface

Andriko von Kügelgen, Vikram Alva, and Tanmay A.M. Bharat

Supplemental information

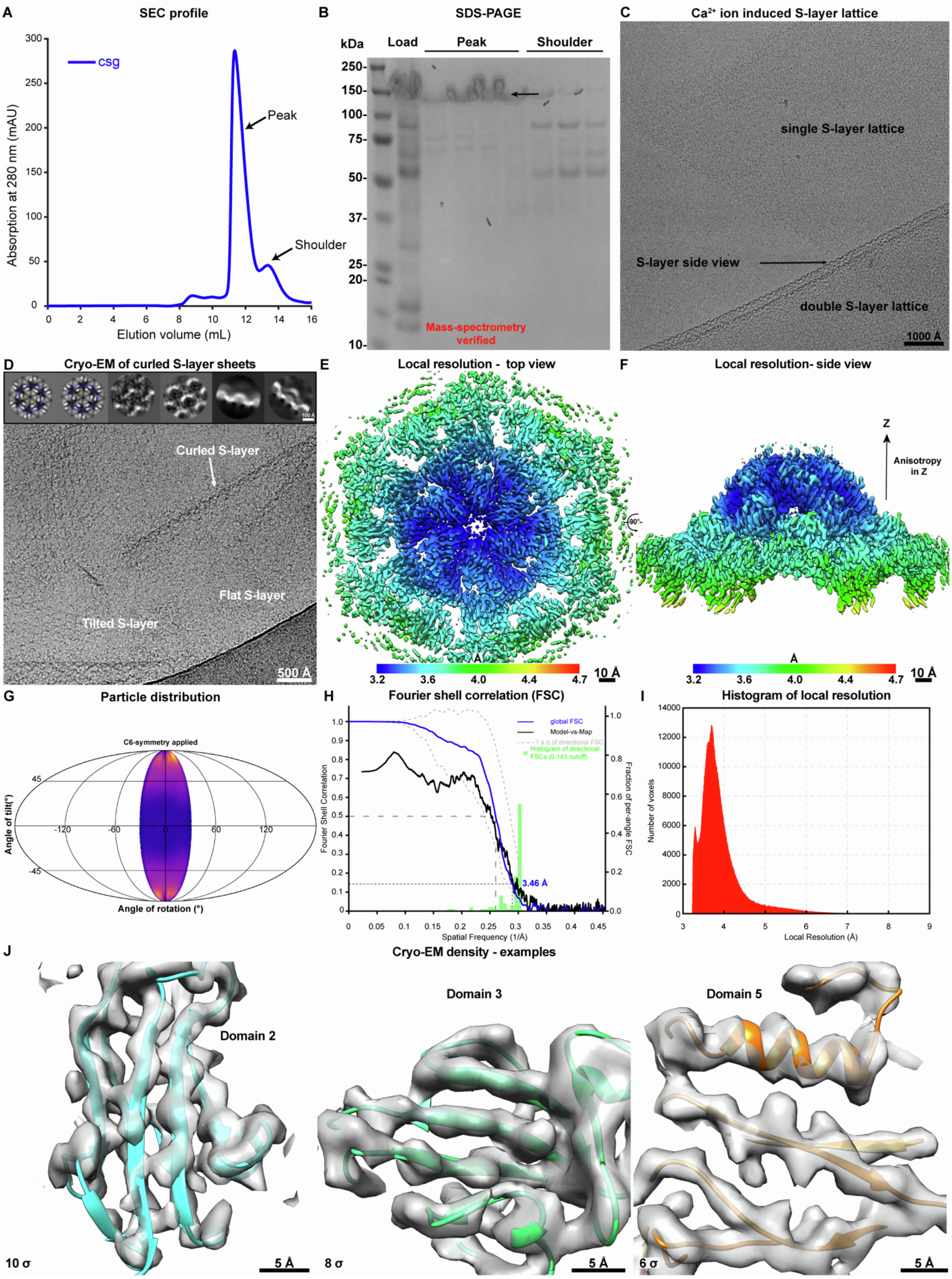

Figure S1. Cryo-EM structure of the csg from two-dimensional sheets (related to Figure 1)

(A) Size-exclusion chromatography (SEC) profile and (B) corresponding SDS-PAGE analysis of purification of csg protein. (C) Incubation of the purified protein with Ca^{2+} led to the formation of two-dimensional sheets (density black), which (D) had variable lattice curvatures. Inset – top, bottom, tilted and side views could be seen in class averages. (E-F) Local resolution estimated in RELION, plotted into the map, shown in two orthogonal orientations. (G) Angular distribution of the particles in the data set show, (H) directional resolution anisotropy, also seen in other two-dimensional crystals. 3D Fourier shell correlation (FSC) as determined by (Tan et al., 2017). Histogram binning size is set to 20. (I) Histogram of local resolutions in voxels of the cryo-EM map. (J) Examples of the csg model built into the reconstructed cryo-EM map of domains D2, D3, and D5 (contour level at the lower left).

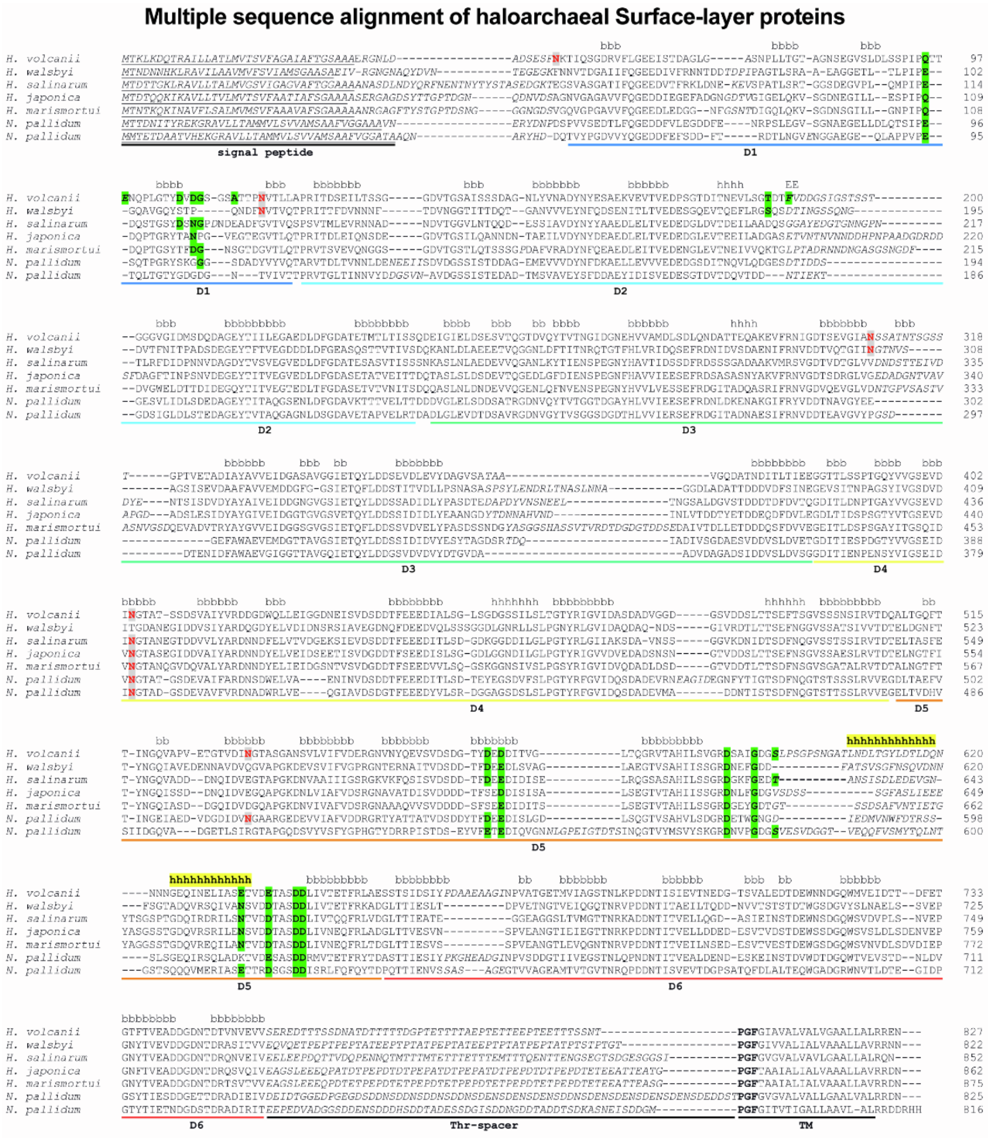

Figure S2. Multiple sequence alignment of haloarchaeal SLPs (related to Figure 2)

Multiple sequence alignment of haloarchaeal SLPs against the csg sequence. An initial alignment was calculated with MSAProbs (Liu et al., 2010) in default settings, and it was subsequently curated manually using pairwise alignments generated with HHpred (pairwise mode in default settings). Residues that could not be aligned are italicized. Domains D1-D6 are marked (underlined with the same color) as in Figure 1B, along with secondary structure observed in our cryo-EM structure (h=α-helix, b=β-strand). D1 is the most divergent in sequence, possibly due to its exposed position and potential role in surface interactions (Sumper et al., 1990). Known glycosylation sites of csg are shown in bold red with grey

highlighting, while conserved cation-binding residues in bold black with green highlighting. A lipid modification is carried out by the archaeosortase A enzyme at the C-terminus after it recognizes and cleaves the tripartite segment, comprising a conserved Pro-Gly-Phe (PGF) motif, a transmembrane helix, and a cluster of basic residues, found at the C-terminus of csg (Abdul Halim et al., 2013). This PGF motif at the C-terminus of the protein after the threonine spacer, important for S-layer anchoring to the membrane, is additionally highlighted (bold black). The experimentally characterized signal peptide of *H. volcanii* and the signal peptides predicted using SignalP5.0 (Almagro Armenteros et al., 2019) for the other SLPs are highlighted. The α-hairpin in D5 that forms a prominent trimeric interface is shown in black bold with yellow highlighting. The NCBI accession IDs of the sequences in the alignment are provided in the legend of Figure S3.

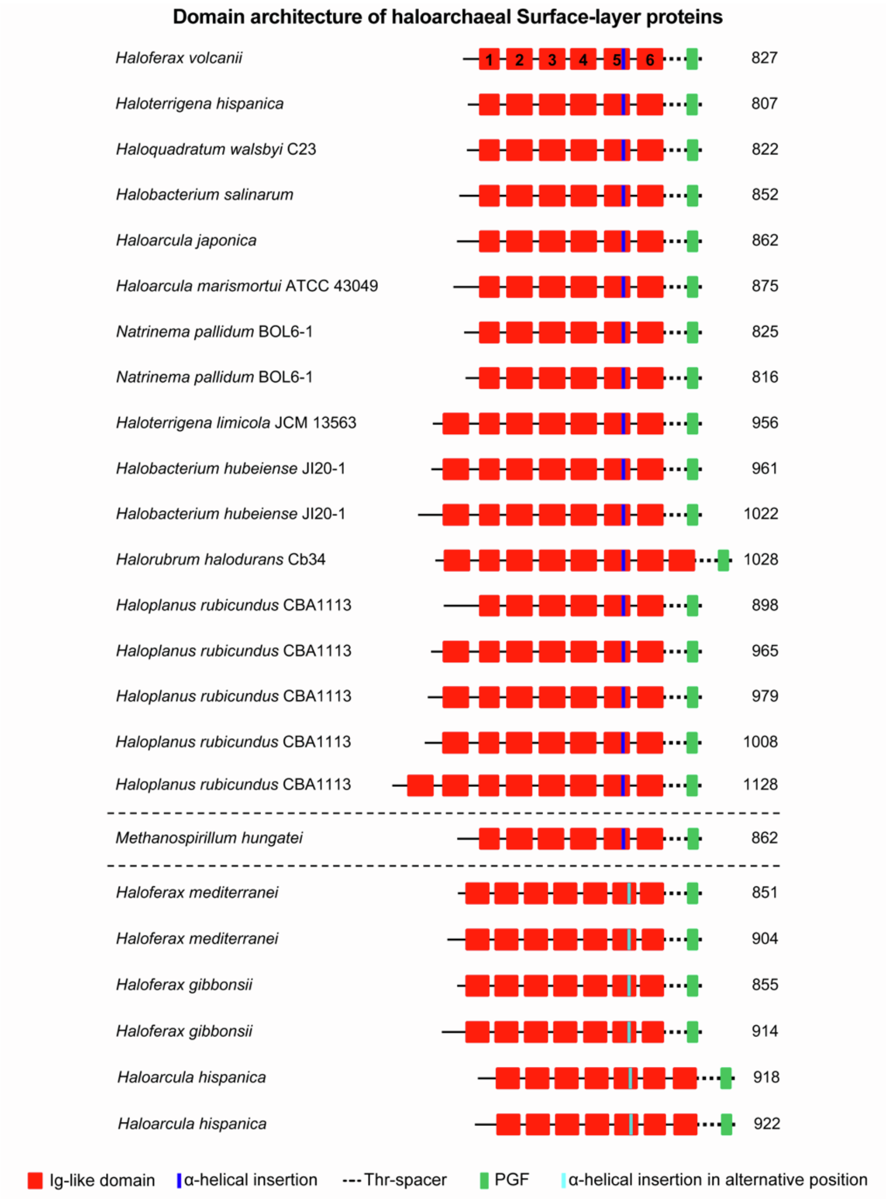

Figure S3. Domain organization of haloarchaeal SLPs (related to Figure 2)

Schematic domain diagrams of haloarchaeal SLPs are shown with the Ig-like domains shown as red boxes. The α-hairpin motif seen in D5 of csg is conserved and found in exactly one domain of other SLPs, usually the penultimate domain before the membrane anchor. This suggests that the cation bound α-helical insertion could be structurally important across haloarchaea, shown here to form the critical trimeric interface in the S-layer lattice. In more divergent haloarchaeal SLPs, such as the ones from *Haloferax mediterranei* and *Haloarcula hispanica*, this helical insertion is found in a different location, usually also in the penultimate domain before the membrane anchor. The NCBI accession IDs of the shown proteins are:

Haloferax volcanii (WP_013035656.1), *Haloterrigena hispanica* (WP_092934807.1), *Haloquadratum walsbyi* C23 (WP_014555115.1), *Halobacterium salinarum* (WP_012289536.1), *Haloarcula japonica* (WP_049909326.1), *Haloarcula marismortui* ATCC 43049 (WP_049938478.1), *Natrinema pallidum* BOL6-1 (WP_006184325.1, ELY78918.1), *Haloterrigena limicola* JCM 13563 (WP_008013949.1), *Halobacterium hubeiense* JI20-1 (CQH46446.1, WP_082687139.1), *Halorubrum halodurans* Cb34 (WP_094534449.1), *Haloplanus rubicundus* CBA1113 (WP_157969218.1, AXG06270.1, WP_114584258.1, AXG07870.1 – start codon was corrected, WP_114587121.1), *Methanospirillum hungatei* (WP_011449226.1, is not a haloarchaeon but contains putative homologous SLP), *Haloferax mediterranei* (WP_004057339.1, WP_004059902.1), *Haloferax gibbonsii* (WP_049904939.1, WP_049905079.1), and *Haloarcula hispanica* (WP_014040752.1, WP_014040753.1). Some haloarchaeal organisms, e.g., *H. rubicundus*, *H. hispanica*, and *H. mediterranei*, contain multiple putative SLPs.

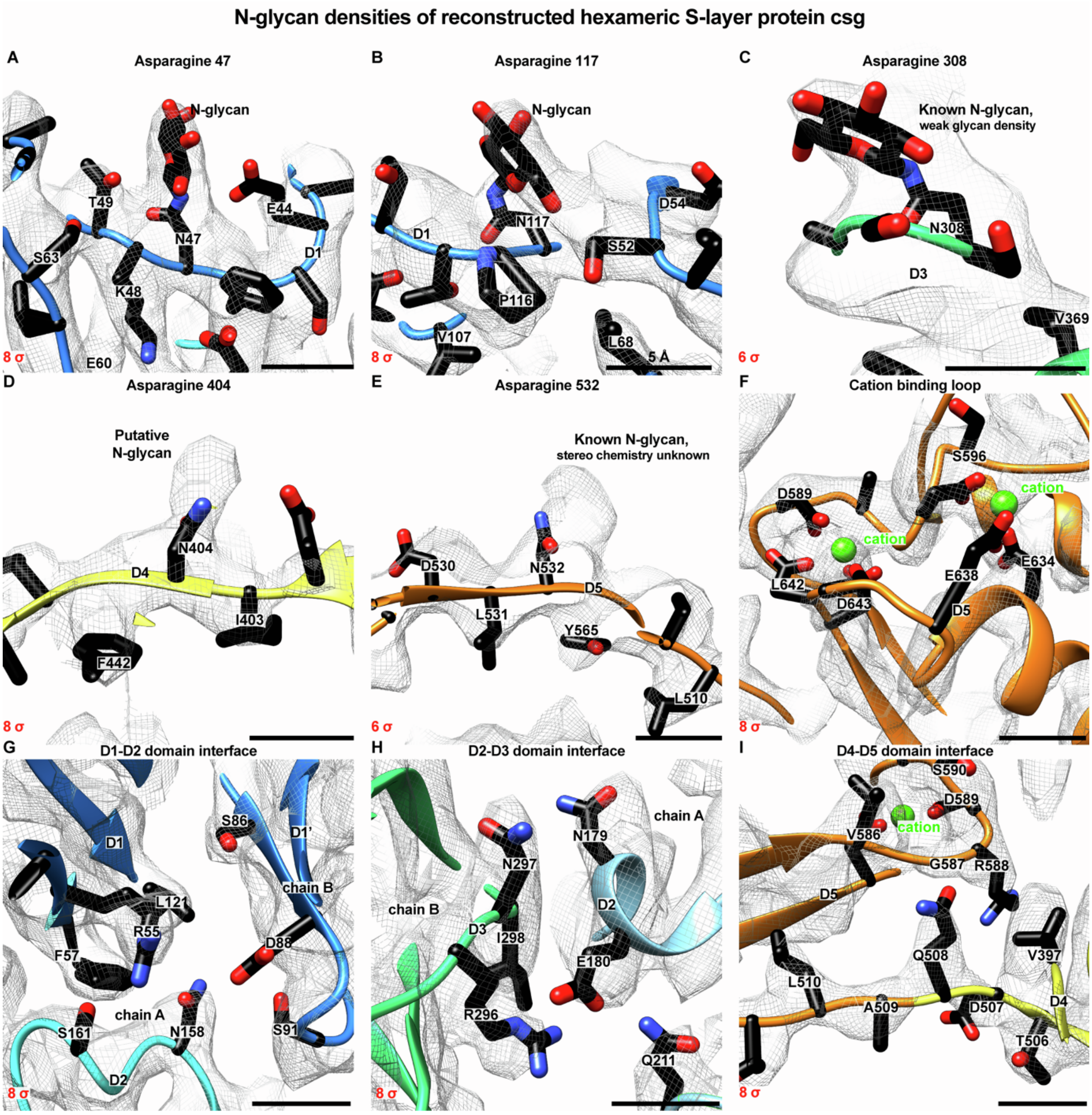

Figure S4. N-linked glycans and cation-binding sites in the csg structure (related to Figures 1 and 3)

(A-E) N-linked glycans resolved in the cryo-EM structure. For N47, N117 and N308 the first sugar has been built to illustrate the glycan location. N532 is a known glycan site, with an undefined stereochemical structure, and was thus not built into the density. Additionally, a large unexplained density was observed at N404, which we predict as hitherto undescribed glycan site (no model built). Only glycan sites that could be resolved and assigned unambiguously are discussed in this study. (F-I) Gallery of still images of the atomic model

shown inside the csg density with F), cation-binding site made of conserved amino acid residues (see Figure S2) and (G-I), domain interfaces.

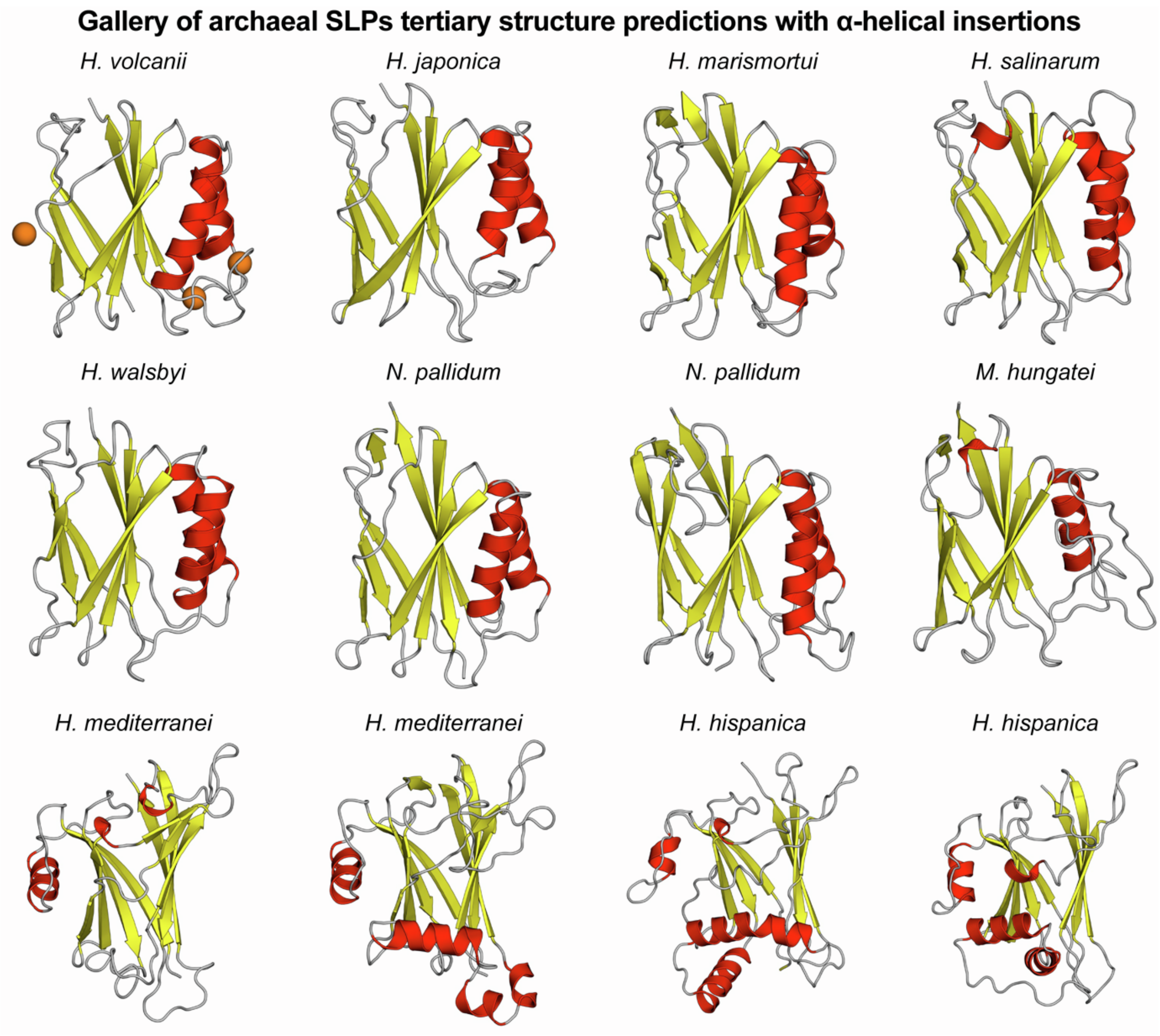

Figure S5. Gallery of SLP domains containing α-helical insertions (related to Figures 1, 2 and 3)

The penultimate domain before the membrane anchor in csg and most haloarchaeal SLPs contains an α-hairpin. In the *H. volcanii* S-layer, this hairpin forms a prominent trimeric interface and homologous regions in other haloarchaeal SLPs are expected to form a similar interface in other S-layers too. Structural models of this domain from several haloarchaeal SLPs were built using the TrRosetta method implemented within the Robetta structure prediction server. In the shown models, α-helices are colored red and β-strands yellow; in the structure of *H. volcanii* D5, bound cations are colored orange. In SLPs that exhibit low pairwise sequence similarities to csg, such as SLPs of *Haloferax mediterranei* and *Haloarcula hispanica*, the α-hairpin occurs in a different location, typically also in the penultimate domain

before the membrane anchor. Outside Haloarchaea, putative SLPs in archaea of the order Methanomicrobiales (e.g., *Methanospirillum hungatei*) also contain such α-helical insertions. The NCBI accession IDs of the proteins are provided in the legend of Figure S3.

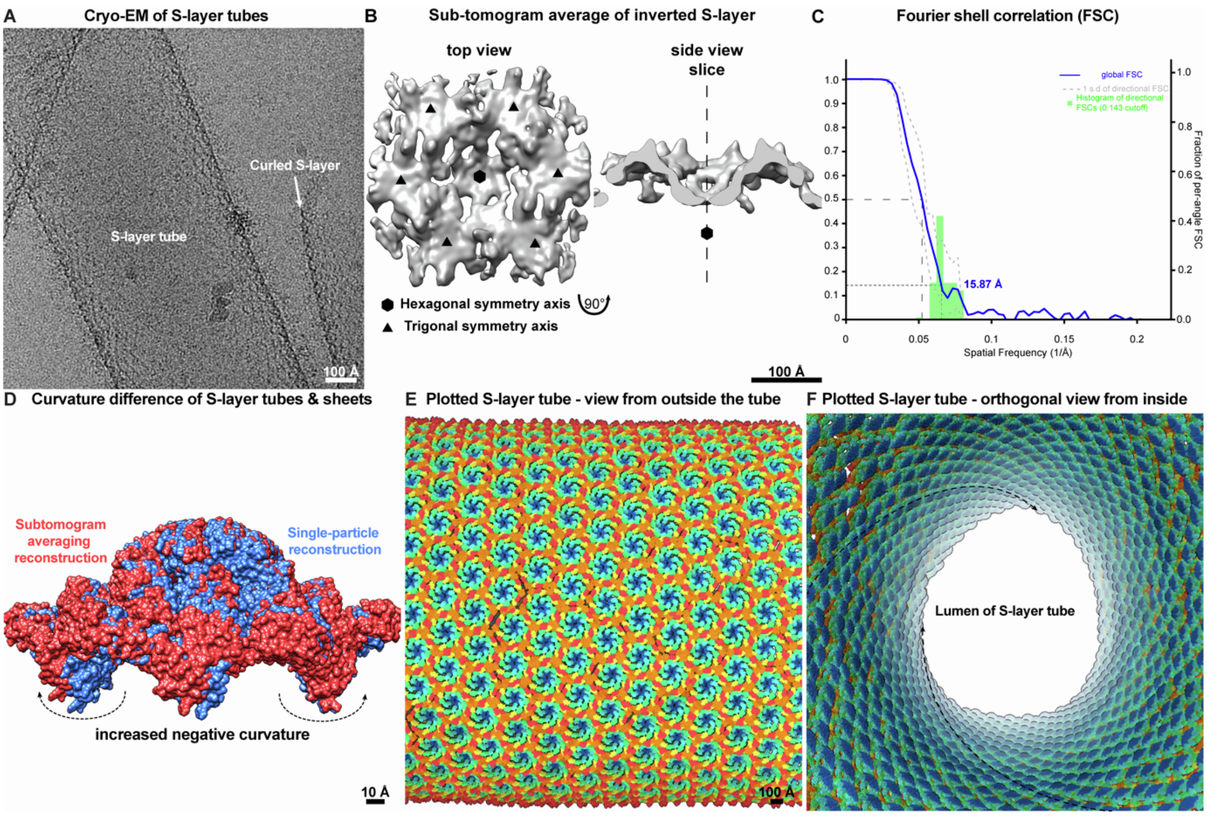

Figure S6. Subtomogram averaging structure of *in vitro* tubes of csg (related to Figure 3)

(A) Cryo-EM image of an *in vitro* S-layer tube. (B) Subtomogram averaging shows an inverted S-layer with a hexameric arrangement. (C) 3D Fourier shell correlation (FSC) with directional resolution anisotropy as described in (Tan et al., 2017). Histogram binning size is set to 7. (D) Comparison of the 15.8 Å resolution subtomogram averaging structure from tubes with the single-particle cryo-EM structure of sheets shows deviation in the D5-D6 region. (E) The subtomogram averaging map plotted onto the surface of the tube, view from the outside, E and inside the tube, F.

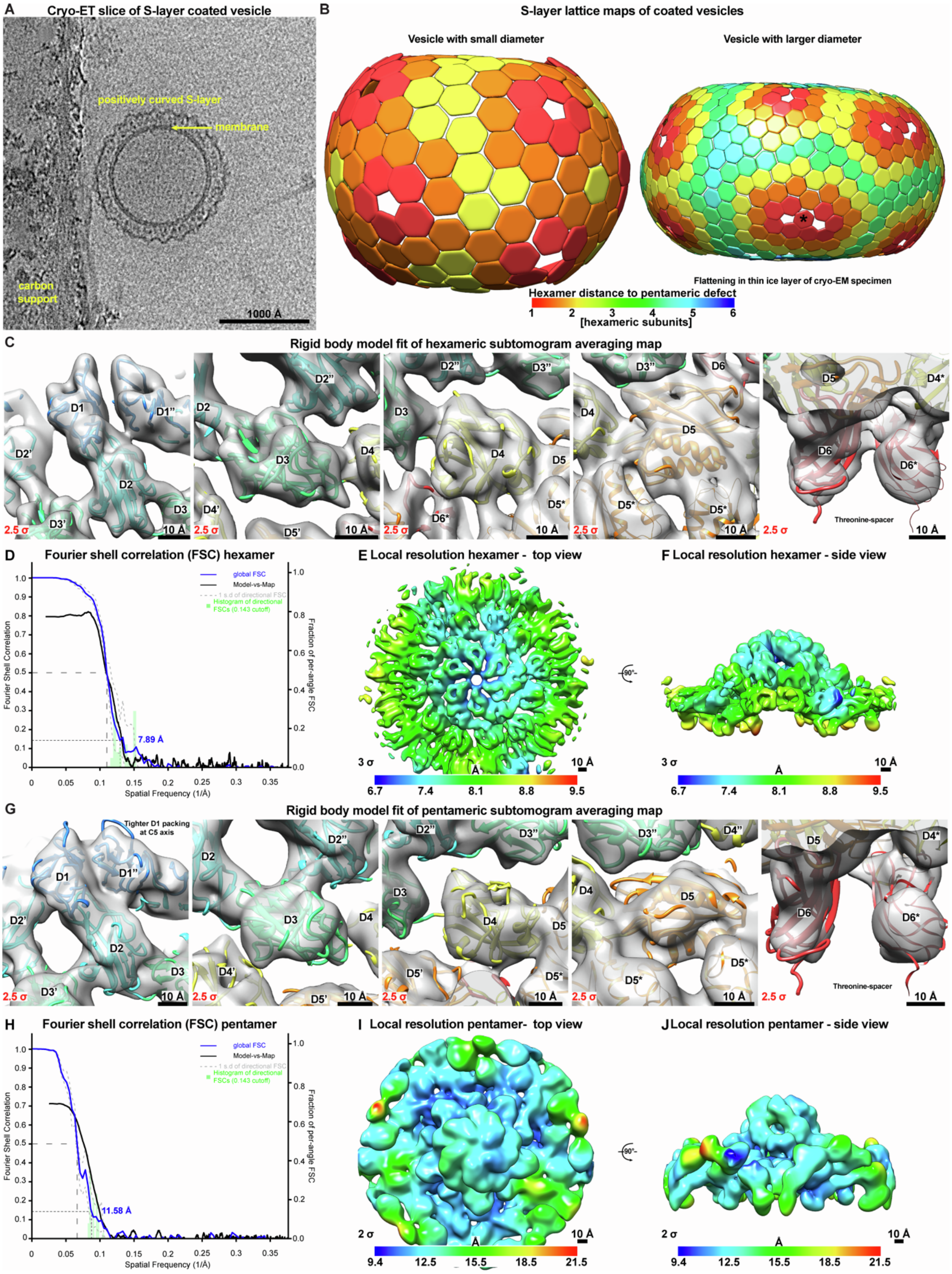

Figure S7. Subtomogram averaging structures of the csg hexamers and pentamers on native membranes (related to Figure 4)

(A) Cryo-ET slice of an isolated S-layer coated membrane vesicle of *H. volcanii* used for subtomogram averaging. (B) Lattice maps (Qu et al., 2018) of the three-dimensional coordinates and orientations of hexameric S-layer positions are colored in a red to blue scale depending on their distance to pentameric defects. A potential heptagon adjacent to pentameric defects is denoted with * (Deatherage et al., 1983). (C) Rigid body model fit of all six domains of the csg model into the hexameric subtomogram averaging map (contour level lower left panel, ‘, ‘’ and * denote domains from other csg monomers). (D) 3D Fourier shell correlation (FSC) of the hexameric subtomogram averaging map as determined by (Tan et al., 2017). Histogram binning size is set to 10. (E-F) Local resolution estimated in RELION, plotted into the hexameric subtomogram averaging reconstruction (contour level lower left panel). (G) Rigid body model fit of all five domains of the csg model into the pentameric subtomogram averaging map. (H) 3D Fourier shell correlation (FSC) of the pentameric subtomogram averaging map as determined by (Tan et al., 2017). Histogram binning size is set to 10. (I-J) Local resolution estimated in RELION, plotted into the pentameric subtomogram averaging reconstruction (contour level lower left panel).

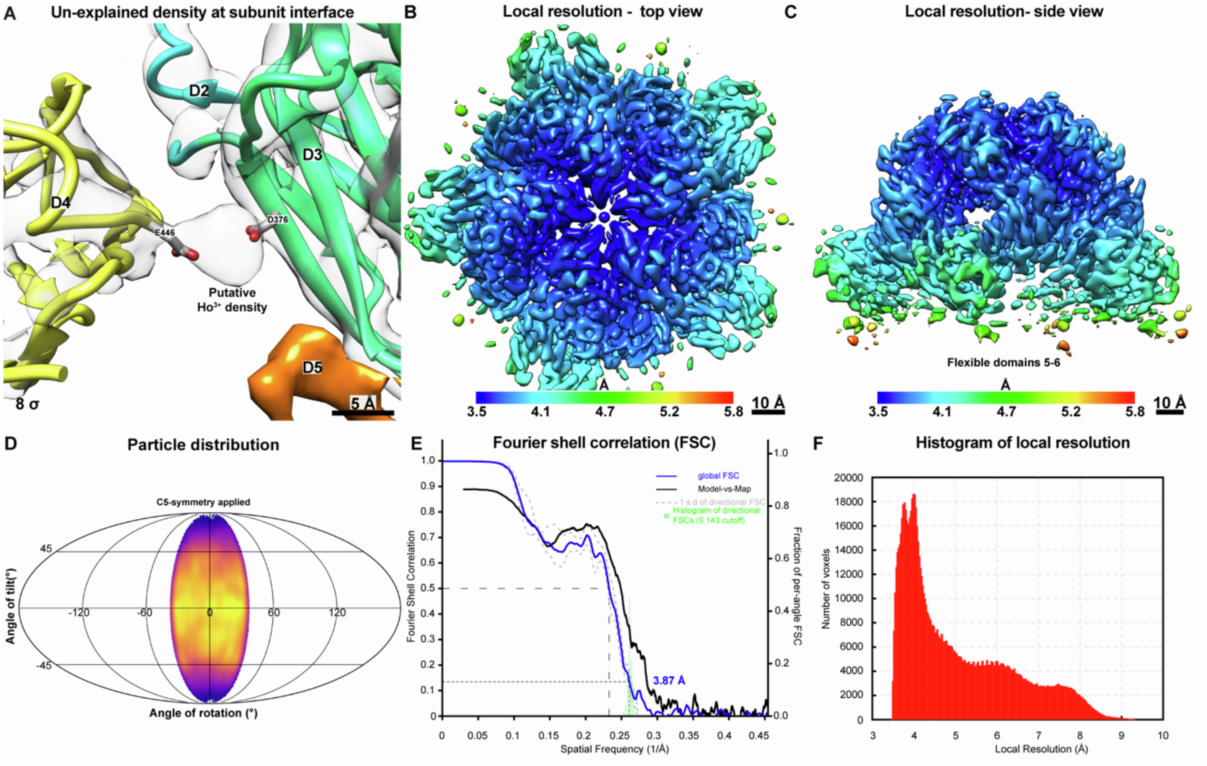

Figure S8. Cryo-EM single particle structure of the csg *in vitro* reconstituted pentamer (related to Figure 6)

(A) Unexplained strong density in the pentamer cryo-EM map, not observed in the hexamer map, possibly from Ho^{3+} ions, since no Ca^{2+} was used for the preparation of this specimen. (B-C) Local resolution estimated in RELION, plotted onto the map. (D) Angular distribution of the particles in the data set. (E) 3D Fourier shell correlation (FSC) as determined by (Tan et al., 2017). Histogram binning size is set to 16. (F) Histogram of local resolutions in voxels of the cryo-EM map.

Table S1. Cryo-EM data collection, refinement and validation statistics. (related to Figures 1 & 6)

	#csg hexamer (EMD-13634) (PDB 7PTR)		#csg pentamer (EMD-13638) (PDB 7PTU)
**Data collection and processing**			
Microscope	Titan Krios G3	Titan Krios G3	Titan Krios G3
Magnification	81,000	81,000	81,000
Voltage (kV)	300	300	300
Electron exposure ($e^-/Å^2$)	49	51.44	53.45
Slit width (eV)	20	20	20
Detector	K3 (Gatan)	K3 (Gatan)	K3 (Gatan)
Defocus range (µm)	-1 to -3	-1 to -3	-1 to -3
Acquisition Mode	Super-resolution	Super-resolution	Super-resolution
Pixel size (Å)	0.55	0.55	0.55
AFIS[&] Mode	Yes	Yes	Yes
Micrographs collected	6,437	12,031	11,871
Micrographs used	5,875	10,906	11,357
**Data processing**			
Software reconstruction	RELION3.1(Zivanov et al., 2020)	RELION3.1(Zivanov et al., 2020)	RELION3.1(Zivanov et al., 2020)
Software picking	TOPAZ(Bepler et al., 2019)/RELION3.1	TOPAZ(Bepler et al., 2019)/RELION3.1	RELION3.0
Initial particle images (no.)	3,747,186	6,811,183	1,773,652
Final particle images (no.)	1,087,798		382,105
Rescaled Box-size Class2D (px)	100 x 100		50 x 50
Rescaled Box-size Class3D (px)	100 x 100 x 100		320 x 320 x 320
Final Box-size (px)	400 x 400 x 400		320 x 320 x 320
Pixel size final reconstruction (Å)	1.1		1.1
Symmetry imposed	C6		C5
Map resolution (Å)	3.46		3.87
FSC threshold	0.143		0.143
Map resolution range (Å)	3.23 - 4.74		3.49 - 8.11
Map sharpening *B* factor ($Å^2$)	-143.26		-170.68
3DFSC sphericity[#]	0.925		0.815
**Model Refinement**			
Initial model used (PDB code)	None		None
Software	PHENIX(Liebschner et al., 2019)		PHENIX(Liebschner et al., 2019)
Model resolution (Å)	3.8		4.0
FSC threshold	0.5		0.5
Model composition			

Non-hydrogen atoms	31,710	17,035
Protein residues	4,350	4,350
Ligand residues	18	5
Ions	18	0
B factors (Å^2)		
Protein	128.24	211.75
Ligand	106.37	153.19
R.m.s. deviations		
Bond lengths (Å)	0.002	0.003
Bond angles (°)	0.454	0.787
Validation		
MolProbity score	1.55	1.80
Clashscore	6.91	8.94
Poor rotamers (%)	0.00	0.26
Cβ outliers (%)	0.00	0.00
CABLAM outliers (%)	1.94	3.62
EMRinger Score	1.24	1.51
Ramachandran plot		
Favored (%)	97.03	95.34
Allowed (%)	2.97	4.66
Disallowed (%)	0.00	0.00

[&] AFIS: Aberration Free Imaging Shift Mode.

[#] 3D-FSC sphericity as determined by the methods described in (Tan et al., 2017).

Table S2. Cryo-ET data collection, refinement and validation statistics. (Related to Figures 3 & S6)

	#csg S-layer tube inverted (EMD-13639)		# csg vesicle hexamer (EMD-13637) (PDB 7PTT)	# csg vesicle pentamer (EMD-13632) (PDB 7PTP)
**Data collection and processing**				
Microscope	Titan Krios G3	Titan Krios G3	Titan Krios	
Magnification	64,000	64,000	105,000	
Voltage (kV)	300	300	300	
Total Electron exposure ($e^-/Å^2$)	90	123	120	
Slit width (eV)	20	25	20	
Detector	K2 Summit	K2 Summit	K2 Summit	
Defocus range (µm)	-2 to -5	-2.5 to -3.5	-1.5 to -3.75	
Pixel size (Å)	2.238	2.238	1.327	
Tilt-series increment	±3°	±3°	±3°	
Tilt-series scheme	bi-directional	dose-symmetric[&]	dose-symmetric[&]	
Tilt-series range	±60°	±60°	±60°	
Tilt-series collected	33	14	172	
Tilt-series used	12	12	127	
**Data processing**				
Software tilt-series alignment	IMOD(Kremer et al., 1996)		IMOD(Kremer et al., 1996)	IMOD(Kremer et al., 1996)
Software final reconstruction	RELION3.1(Zivanov et al., 2020)		RELION3.1(Zivanov et al., 2020)	RELION3.1(Zivanov et al., 2020)
Initial particle images (no.)	25,843		83,713	3,495
Final particle images (no.)	25,843		53,063	1,640
Rescaled Box-size Class3D (px)	128 x 128 x 128		96 x 96 x 96	192 x 192 x 192
Final Box-size (px)	128 x 128 x 128		192 x 192 x 192	192 x 192 x 192
Pixel size final reconstruction (Å)	2.238		1.327	1.327
Symmetry imposed	C2		C6	C5
Map resolution (Å)	15.87		7.968	11.58
FSC threshold	0.143		0.143	0.143
Map resolution range (Å)	13 - 24		6.7-9.5	9.4-21.5
Map sharpening *B* factor ($Å^2$)	*none*		*none*	*none*
3DFSC sphericity[#]	0.953		0.969	0.985
**Model Refinement**				
Initial model used (PDB code)	N.D.		7PTR	7PTR

Software	-	PHENIX(Liebschner et al., 2019)	PHENIX(Liebschner et al., 2019)
Model resolution (Å)	N.D.	9.1	13.1
FSC threshold	N.D.	0.5	0.5
Model composition			
Non-hydrogen atoms	N.D.	31488	26240
Protein residues	N.D.	4350	2625
R.m.s. deviations			
Bond lengths (Å)	N.D.	0.003	0.003
Bond angles (°)	N.D.	0.616	0.608
Validation			
MolProbity score	N.D.	1.99	2.00
Clashscore	N.D.	14.77	15.06
Poor rotamers (%)	N.D.	0.00	0.00
Cβ outliers (%)	N.D.	0.00	0.00
CABLAM outliers (%)	N.D.	3.47	3.47
Ramachandran plot			
Favored (%)	N.D.	95.41	95.41
Allowed (%)	N.D.	4.59	4.59
Disallowed (%)	N.D.	0.00	0.00

[&] Dose-symmetrical tilt-scheme as described in (Hagen et al., 2017).

[#] 3DFSC sphericity as determined by the methods described in (Tan et al., 2017).

N.D. not determined

Table S3: Archaeal strains used in this study (related to STAR methods).

Strain or plasmid	Description or construction	Source or reference
**Strains**		
Haloferax volcanii		
H-26	*H. volcanii* Δ*pyrE2*	(Allers et al., 2004)
