# Supplementary material for: Complete atomic structure of a native archaeal cell surface
Source: Cell Rep. 2021 Nov 23;37(8):110052. doi: 10.1016/j.celrep.2021.110052 (PMC8640222; doi:10.1016/j.celrep.2021.110052)
Supplement: Document S2. Article plus supplemental information [file mmc5.pdf]

Cell Reports

Article

Complete atomic structure of a native archaeal cell surface

Graphical abstract

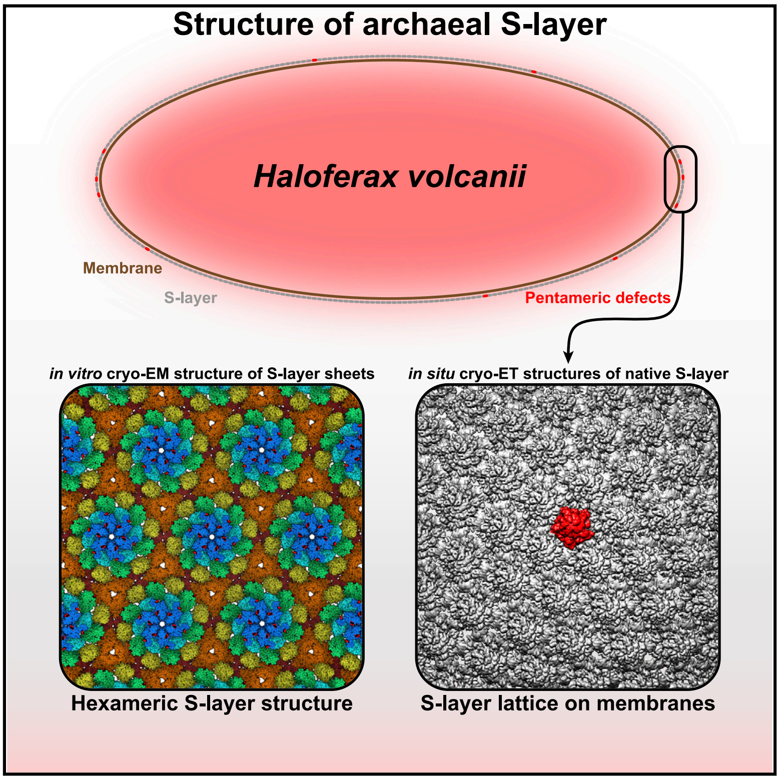

Authors

Andriko von Kügelgen, Vikram Alva, Tanmay A.M. Bharat

Correspondence

tanmay.bharat@path.ox.ac.uk

In brief

von Kügelgen et al. show, using *in situ* cryo-EM, how *Haloferax volcanii* archaea are nearly perfectly coated by a hexameric surface layer with pentameric defects, made of a protein with repeated immunoglobulin-like domains. Structure- and sequence-based bioinformatics reveal widespread prokaryotic conservation of these domains, with implications on evolution and fundamental archaeal biology.

Highlights

- Cryo-EM reveals the structure of the hexameric S-layer of *Haloferax volcanii* archaea
- Immunoglobulin-like domains are widely conserved in archaeal S-layers
- Cryo-ET shows how an S-layer made of hexamers and pentamers coats curved membranes
- Atomic-level description of a native archaeal cell surface is shown

von Kügelgen et al., 2021, Cell Reports *37*, 110052
November 23, 2021 © 2021 The Author(s).
https://doi.org/10.1016/j.celrep.2021.110052

CellPress

CellPress
OPEN ACCESS

Article

Complete atomic structure of a native archaeal cell surface

Andriko von Kügelgen,[1] Vikram Alva,[2] and Tanmay A.M. Bharat[1,3,4,*]
[1]Sir William Dunn School of Pathology, University of Oxford, Oxford OX1 3RE, UK
[2]Department of Protein Evolution, Max Planck Institute for Developmental Biology, Max-Planck-Ring 5, Tübingen 72076, Germany
[3]Structural Studies Division, MRC Laboratory of Molecular Biology, Francis Crick Avenue, Cambridge CB2 0QH, UK
[4]Lead contact
*Correspondence: tanmay.bharat@path.ox.ac.uk
https://doi.org/10.1016/j.celrep.2021.110052

SUMMARY

Many prokaryotic cells are covered by an ordered, proteinaceous, sheet-like structure called a surface layer (S-layer). S-layer proteins (SLPs) are usually the highest copy number macromolecules in prokaryotes, playing critical roles in cellular physiology such as blocking predators, scaffolding membranes, and facilitating environmental interactions. Using electron cryomicroscopy of two-dimensional sheets, we report the atomic structure of the S-layer from the archaeal model organism *Haloferax volcanii*. This S-layer consists of a hexagonal array of tightly interacting immunoglobulin-like domains, which are also found in SLPs across several classes of archaea. Cellular tomography reveal that the S-layer is nearly continuous on the cell surface, completed by pentameric defects in the hexagonal lattice. We further report the atomic structure of the SLP pentamer, which shows markedly different relative arrangements of SLP domains needed to complete the S-layer. Our structural data provide a framework for understanding cell surfaces of archaea at the atomic level.

INTRODUCTION

All prokaryotes interact with their environment through molecules on the cell surface. These cell-surface molecular interactions allow them to locate nutrients, recognize threats, and adapt to their surroundings (Bharat et al., 2021). For prokaryotic organisms living in vastly varied and often harsh habitats, this need for specialized cell-surface molecules is paramount (Filloux and Whitfield, 2016). The entire outer surface of many bacterial and archaeal cells is covered by a two-dimensional array of proteinaceous molecules, a supramolecular structure known as the surface (S-)layer (Sára and Sleytr, 2000). S-layers are built by repeated interactions of S-layer proteins (SLPs), which are often the most abundant cellular molecules, representing up to 15% of the entire protein content of a cell (Bharat et al., 2021; Fagan and Fairweather, 2014; Pum et al., 2013). Owing to their plentitude in prokaryotes, estimates place SLPs as one of the most abundant classes of proteins on Earth (Pum et al., 2013). Despite this, at the fundamental molecular level, relatively little is known structurally about these enigmatic sheet-forming proteins (Bharat et al., 2021).

While some progress has recently been made on understanding bacterial S-layers in a series of structural biology studies (Baranova et al., 2012; Bharat et al., 2017; Fioravanti et al., 2019; von Kügelgen et al., 2020), S-layers from archaea are less understood at the atomic level. Thus far, high resolution atomic structural data have been reported only for the C-terminal repeat segment of the SLP from *Methanosarcina acetivorans* (Arbing et al., 2012). This segment exhibits two structurally similar β-sandwich folds that is also found in envelope proteins of some eukaryotic RNA viruses. Although the availability of atomic-level structural data on archaeal S-layers is limited, several elegant structural studies have revealed their symmetric organization, providing clues to the constitution and arrangement of these paracrystalline arrays (Arbing et al., 2012; Deatherage et al., 1983; Kessel et al., 1988; Taylor et al., 1982; Trachtenberg et al., 2000). Additionally, some recent tomographic studies have shown the overall arrangement of S-layer lattices at molecular resolution in some archaeal species (Gambelli et al., 2019; Li et al., 2018).

In archaea, S-layer genes are usually the highest expressed, and the corresponding SLPs are, in turn, the highest copy number proteins (Albers and Meyer, 2011; Li et al., 2018). Being the outermost surface in many archaeal cells (Klingl, 2014), S-layers mediate important interactions with their environment, often acting as a molecular sieve regulating the transport of materials to the cell (Li et al., 2018). Additionally, they fulfill several critical cellular functions, including protection from predators and phages (Sára and Sleytr, 2000; Zink et al., 2019), maintenance of a pseudo (or quasi)-periplasmic space outside the cell membrane (Albers and Meyer, 2011), preservation of cell shape (Bharat et al., 2021; Zink et al., 2019), stabilization of the cell membrane (Abdul-Halim et al., 2020), and biomineralization (Kish et al., 2016; Milojevic et al., 2019). In some archaea, S-layers have also been implicated in processes linked to the cell cycle (Albers and Meyer, 2011) as well as to cell division and

© 2021 The Author(s).
This is an open access article under the CC BY license (http://creativecommons.org/licenses/by/4.0/).

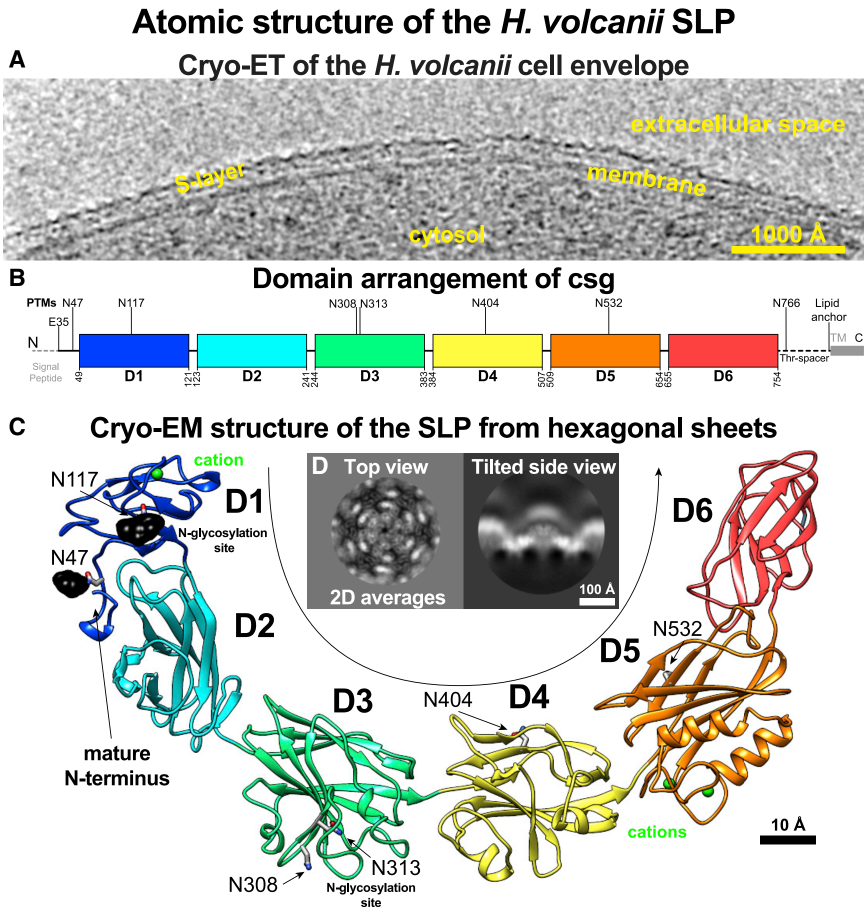

Figure 1. Atomic structure of the *H. volcanii* SLP csg

(A) Cryo-ET slice through the surface of an *H. volcanii* cell. Density (black) corresponding to the S-layer is marked, showing a tight association with the membrane.

(B) The csg protein contains six domains (D1–D6), colored blue to red in the schematic. Sites of post-translational modifications including the signal-peptide processing, the known and expected protein glycosylation, the O-glycosylated threonine spacer region, and the lipid anchor are shown.

(C) Cryo-EM structure of csg shows that D1–D6 adopt Ig-like folds. The locations of the N-linked glycans are shown in black, and bound cations are shown as green spheres.

(D) Two-dimensional class averages of the sheet-like specimen used for cryo-EM structure determination. Characteristic top and side views are visible.

elongation machinery (Zink et al., 2019). Consequently, understanding the structure, organization, and assembly of S-layers is vital to understanding the basic biology of archaea.

H. volcanii is a genetically tractable model haloarchaeon found in hypersaline environments of the Dead Sea (Mullakhanbhai and Larsen, 1975). It forms pleiomorphic cells surrounded by a proteinaceous S-layer (Figure 1A) with hexagonal symmetry (Kessel et al., 1988), which extends up to ~125 Å away from the cell surface (Sumper et al., 1990). The *H. volcanii* S-layer is essential for cell survival and plays vital roles in cell shape maintenance, mating, and many aspects of the cell cycle (Abdul-Halim et al., 2020; Shalev et al., 2017; Tamir and Eichler, 2017). This S-layer consists of repeating units of a single 827-residue SLP called cell-surface glycoprotein (csg) (Figure 1B), which is highly enriched in negatively charged amino acid residues, highlighting the adaptation of *H. volcanii* to hypersaline environments. While homologs of csg have been characterized and shown to form S-layers with hexagonal lattices in several other haloarchaea (Lechner and Sumper, 1987; Lu et al., 2015; Wakai et al., 1997), csg has, in particular, been extensively studied with electron microscopy (EM) (Kessel et al., 1988) and used as a prominent model system for investigating protein modifications in archaea, owing to extensive post-translational modifications (Kaminski et al., 2013; Kandiba and Eichler, 2014; Kandiba et al., 2016; Konrad and Eichler, 2002; Parente et al., 2014). An archaeosortase-dependent lipid modification occurs at the C terminus of csg after cleavage of a tripartite segment, comprising a conserved Pro-Gly-Phe (PGF) motif, a transmembrane (TM) helix, and a cluster of basic residues (Abdul Halim et al., 2013). Several cryo-electron-microscopy (cryo-EM) studies of *H. volcanii* cell envelopes have shown that the S-layer is tightly anchored to the cell membrane (Sivabalasarma et al., 2021; Tamir and Eichler, 2017) through these lipid modifications (Konrad and Eichler, 2002) (Figures 1A and 1B). Furthermore, the N-glycosylation of the *H. volcanii* S-layer has also been intensively analyzed to investigate cell mating (Shalev et al., 2017), a process haloarchaea employ for horizontal gene transfer across cells (Rosenshine et al., 1989). Such mating cells are connected by cell-cell bridges enveloped by a continuous S-layer lattice (Sivabalasarma et al., 2021).

To understand the fascinating aspects of archaeal S-layers, including specifically those of *H. volcanii* introduced above, we present the atomic structure of the S-layer from *H. volcanii.* By visualizing the S-layer *in situ*, directly in its native environment, we report the structure of the hexagonal S-layer that coats variably curved cellular membranes. We further report the structure of SLP pentamers, showing how the lattice is closed around cells in three dimensions by pentameric defects. Structures of S-layer hexamers and pentamers at different lattice curvatures and states allow us to exhibit an atomic-resolution description of the entire outermost surface of *H. volcanii*.

RESULTS

Cryo-EM structure of the csg hexamer

To understand cell-surface organization in haloarchaea, we aimed to resolve the atomic structure of the model S-layer from *H. volcanii.* To this end, we purified native csg protein

CellPress
OPEN ACCESS

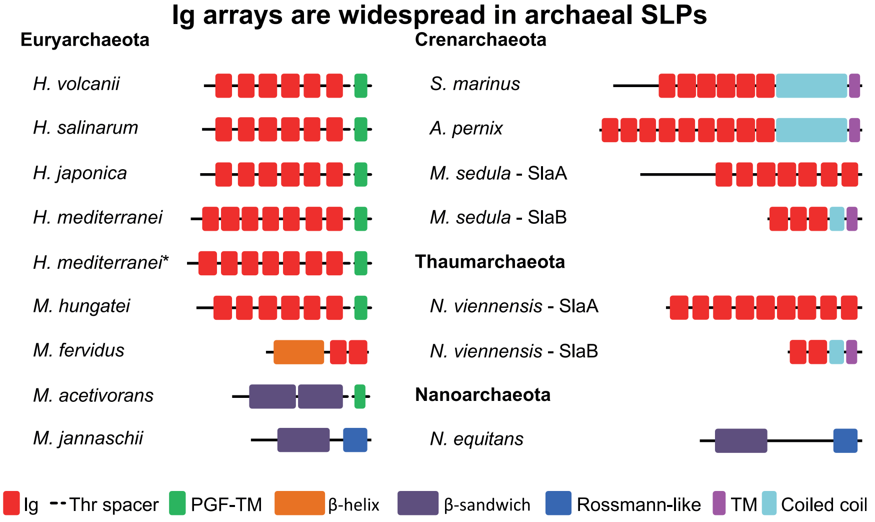

Figure 2. Sequence- and structure-prediction-based analysis reveals underlying domain organization of archaeal SLPs

The multi-domain Ig-like arrangement observed in csg is found across different archaeal phyla. In SLPs outside haloarchaea, Ig-like arrays are typically found in conjunction with coiled-coil segments or a single-stranded right-handed β-helix and a transmembrane (TM) anchor. Some SLPs contain a different β-sandwich domain and a C-terminal csg-like membrane anchor preceded by a conserved PGF motif (PGF-TM) or a putative small Rossmann-like anchor. The SLP of *M. acetivorans* contains two homologous repeat regions. The structure of the C-terminal repeat region has been characterized (PDB: 3U2H), and it exhibits two structurally similar β-sandwich folds. More details are provided in Figure S3. We note that we were unable to confidently assign some highly divergent parts of the of the sequences in SLPs of *M. jannaschii*, *S. marinus*, *M. sedula* (SlaA), and *N. equitans*.

from *H. volcanii* cells and incubated it with Ca^{2+} ions (Figures S1A–S1C). In the resulting mixture, we observed S-layer-like sheets with variable curvature (Figure S1D), showing characteristic top, tilted, and some side views (Figure S1D). We used single-particle cryo-EM data analysis to resolve a global 3.5-Å resolution structure of the hexameric repeating unit of the S-layer within the sheets (Figures S1E–S1J; Table S1) and used it to build an atomic model of csg (Figures 1C and 1D; Video S1; Method details).

The atomic model revealed that each csg monomer assumes an overall sickle shape and is organized into six β-rich immunoglobulin (Ig)-like domains (D1–D6 from hereon) (Figure 1C), of which D1 is highly divergent in sequence among other haloarchaea (Sumper et al., 1990) (Figure S2). Sensitive homology searches and tertiary structure prediction revealed that such arrays of Ig-like folds are widespread across archaeal SLPs (Figure 2), suggesting shared principles of S-layer organization among many archaea. In particular, all analyzed haloarchaeal SLPs had domain compositions similar to csg, with some additional N- or C-terminal Ig-like domains (Figure S3). Such Ig-like domain arrays were also detected, typically in conjunction with other domains, in several non-haloarchaeal SLPs (Figure 2), indicating that these archaea may share similar cell-surface features. For instance, the two putative SLP subunits of *Nitrososphaera viennensis*, SlaA and SlaB, contain multiple Ig domains (Figure 2). However, some non-haloarchaeal SLPs, such as those of *Methanocaldococcus jannaschii* and *Nanoarchaeum equitans*, were predicted to contain no Ig-like domains and instead contain multiple copies of a distinct β-sandwich domain, which is also found in the structurally characterized SLP of *M. acetivorans* (Arbing et al., 2012).

In our atomic structure, we observed additional cryo-EM densities at several asparagine residues, which we assigned to known glycosylation sites (Kaminski et al., 2013; Kandiba and Eichler, 2014; Kandiba et al., 2016; Parente et al., 2014) (Figures 1C and S4A–S4E). An N-linked glycan close to the N terminus (E35) of the mature protein on residue N47 probably shields the N terminus from proteolytic cleavage by steric hindrance, in line with previous biochemical experiments (Tamir and Eichler, 2017). At several locations in the protein, unexplained densities were observed within the fold of the protein, coordinated to negatively charged amino acid residue side chains. Given the known dependence of csg on Ca^{2+} for lattice assembly (Cohen et al., 1991; Rodrigues-Oliveira et al., 2019) and the high concentration of Mg^{2+} in the medium, we expected that these densities correspond to Ca^{2+} or Mg^{2+} cations (Figure S4F).

The csg monomers were packed into the sheet as hexamers (Figure 3A; Video S1). The 6-fold symmetry axis of the hexamer contains repeated interactions of D1 around the central pore, which is ~13 Å wide (Figure 3B). A glycan on N117 is located close to the hexameric interface, creating a dense network of glycans around this site, further obscuring access of molecules to the narrow pore. The pore is lined with negatively charged residues bound to metal ions, likely Ca^{2+} or Mg^{2+}, which, in agreement with previous studies, appear to stabilize the hexameric interface critical for lattice formation (Cohen et al., 1991). Because of the sickle shape of the csg monomer, there are repeated intra-hexamer domain interactions between each monomer involving all six domains (Figures 3A and S4G–S4I), resulting in a tight arrangement of the hexamer with multiple protein:protein interfaces.

The nonporous hexagonal S-layer lattice supports flexible curvatures

The csg hexamers are packed into a *p*6 two-dimensional lattice, with inter-hexamer interactions mediated by domains D4, D5, and D6 (Figures 3C and 3D). A conserved α-hairpin motif in D5 forms a prominent trimeric interface (Figure 3C), while stacked D4 and D6 from adjoining hexamers form an extensive dimeric interface (Figure 3D), resulting in a lattice with almost no gaps. Bioinformatic analyses further supported the importance of the α-hairpin motif in D5 that forms the trimeric interface (Figure S3). Many haloarchaeal SLPs with a variable number of Ig-like domains almost always contain only one domain, typically the penultimate domain in the array, with this α-hairpin (Figure S3). The key trimeric D5 inter-hexameric interface is stabilized by

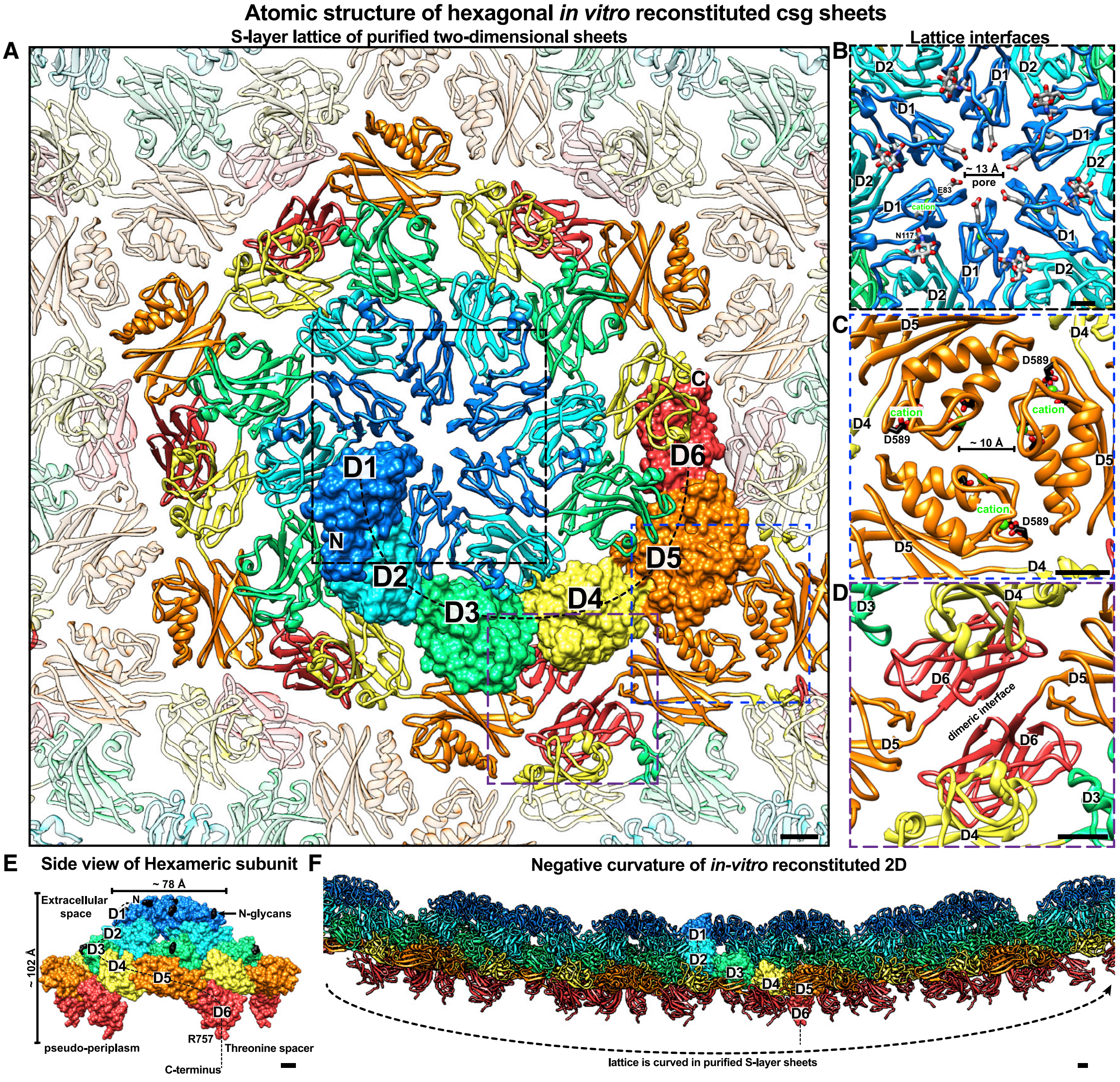

Figure 3. Atomic structure of the hexagonal S-layer lattice

(A) Each csg monomer is arranged into hexamers in a two-dimensional lattice. D1–D6 for one csg monomer are shown in a surface representation.
(B) Magnified view of the hexameric interface (dashed black box in A) reveals glycans and cation-bound residues near the pore in D1.
(C) Magnified view of the trimeric interface (dashed blue box in A) reveals an α-hairpin with bound cations mediating inter-hexameric contacts in D5.
(D) Magnified view of the dimeric interface (dashed purple box in A) shows stacked D4 and D6 mediating a substantial inter-hexameric contact site.
(E) Side view of the csg hexamer, along the plane of the sheet, shows the dome-shaped D1 and D2 structure.
(F) Side view of the lattice. The protein lattice is negatively curved *in vitro*. Scale bars: 10 Å.

several functional divalent cations bound by the α-hairpin of D5, explaining the observed Ca^{2+} dependence of lattice assembly (Cohen et al., 1991). These residues are conserved in haloarchaea (Figure S2), further supporting their importance in lattice assembly. In more divergent haloarchaeal SLPs that share less than 15% pairwise sequence identity with csg, such as the ones of *Haloferax mediterranei* and *Haloarcula hispanica*, the α-hairpin is located in a different position in the domain corresponding to D5 (Figures S3 and S5).

When viewed along the plane of the sheet, D1 and D2 form a dome-shaped structure, with D5 and D6 at the base (Figure 3E). Our reconstituted S-layer sheets displayed a wide variety of lattice curvatures (Figure 3F) and, in extreme cases, curled up into tube-like structures (Figure S6), demonstrating that even though the lattice is tightly packed and nonporous, it can deviate significantly from a planar arrangement, a requirement for coating cellular membranes with variable curvature. To test this hypothesis further, we performed subtomogram averaging (STA) of the tube-like structures and produced a 15.8-Å-resolution structure (Figure S6; Table S2). Fitting of the csg atomic model into the cryo-ET map showed marked deviations in the positions of D5 and D6, suggesting that rearrangement of lattice interfaces in

CellPress
OPEN ACCESS

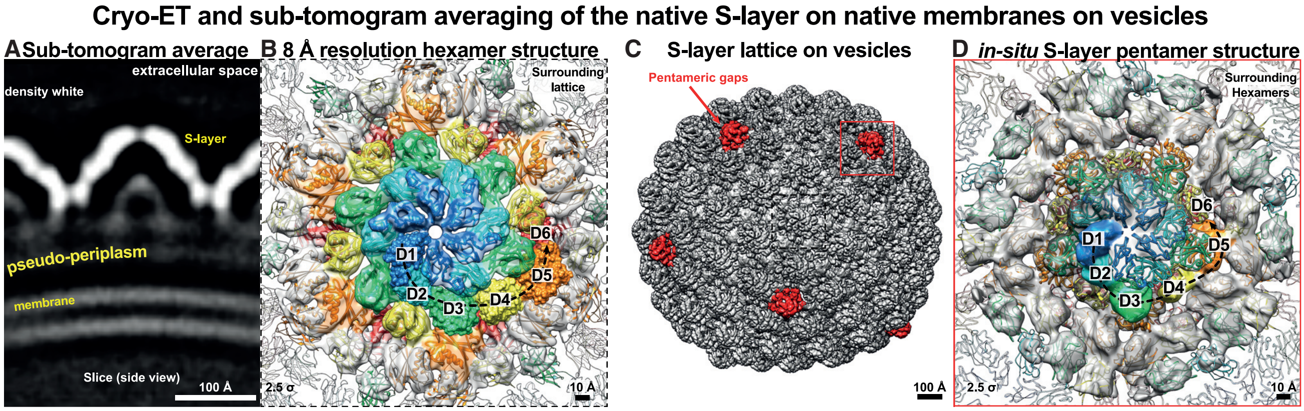

Figure 4. S-layer organization on native membranes

(A) Cryo-ET and STA from vesicles of *H. volcanii* show the characteristic dome-shaped S-layer enclosing the known pseudo-periplasmic space over the membrane.

(B) Despite opposite lattice curvature, the atomic structure of csg could be unambiguously fitted into the 8-Å resolution cryo-ET density (contour level lower left of panel).

(C) Plotting the resolved structure back onto the tomograms of vesicles revealed a nearly continuous lattice with pentameric defects (red).

(D) STA of the pentameric defects showed that these sites contain the same csg protein with D1–D6, fitted in as rigid bodies separately into the 11.5-Å resolution map.

D5 and D6 helps accommodate these variable lattice curvatures (Figure S6).

The native S-layer is nearly continuous with pentameric defects

We next investigated how the *in vitro* reconstituted csg sheets were related to the native S-layer on membranes. To this end, we purified vesicles from *H. volcanii* cultures that have been previously observed (Shalev et al., 2017) and visualized them with cryo-ET. STA of the S-layer lattice from these vesicles resulted in a sub-nanometer-resolution (8.0 Å) structure (Figures 4A, 4B, and S7; Table S2), where all six domains of csg, detected in our single-particle cryo-EM structure (Figures 1 and 3), could be unambiguously resolved. The relative arrangements of the domains in our STA map were nearly identical to those seen in the *in vitro* sheets (Figure 4B). The cryo-ET map on native membranes showed that the dome formed by D1 and D2 faces away from the membrane, meaning that the N-linked glycans described above (Figure 1) point outward away from the cell, where they can play their proposed role in cellular recognition required for key processes such as mating (Shalev et al., 2017).

A notable difference in the S-layer structure on membranes to the *in vitro* specimen was opposite lattice curvature, suggesting that the membrane might play an important role in affecting lattice curvature (Figure 4A). Another striking feature observed in the S-layer lattice on membranes was the nearly perfect continuity of the S-layer around the vesicles (Figure 4C). Rather than having gaps in the lattice, pentameric defects were observed (Figure 4C). We next extracted subtomograms at the positions of these defects and produced an 11.5-Å-resolution STA map (Figures 4D and S7; Video S2). This map showed clear densities for all six csg domains, confirming that the pentameric defects are made of the same csg protein. This ability of csg to close gaps in the lattice leads to a near-perfect coating of cellular membranes that are known to adopt a variety of curvatures in rod-shaped, spherical, and polymorphic cells (Duggin et al., 2015; Sivabalasarma et al., 2021).

To confirm that our results on purified vesicles faithfully represented the situation on cells, we repeated the cryo-ET and STA experiment on whole *H. volcanii* cells and produced lattice maps that showed the same hexagonal lattice with pentameric defects (Figure 5A). The unit cell size, along with the modular arrangement of the cellular S-layer, was the same as the S-layer studied on vesicles (Figure 4A), confirmed by the lattice maps obtained on cells (Figure 5A). In our data from cells, the number of pentamers appeared to be increased in areas of the cell with a sudden change in membrane curvature (Figures 5B and 5C). *H. volcanii* is known to form cells with a variety of shapes (Duggin et al., 2015), and these experiments demonstrate that pentamers of csg are present in the native environment in cells and likely support the maintenance of S-layer coating of membranes with widely different curvatures, while maintaining lattice continuity.

Cryo-EM structure of the csg pentamer

Given the key importance of pentamers in the S-layer lattice, we next set out to resolve an atomic structure of csg pentamers to understand how the SLP changes conformation to incorporate into defects in the lattice. Ho^{3+} ions are known to replace Ca^{2+} ions in proteins, and this property has been effectively used in the past for phasing of SLP crystals for X-ray structure determination (Bharat et al., 2017). Following this strategy, we were able to reconstitute pentamers of csg by incubating the protein with Ho^{3+} ions. Single particles were observed in cryo-EM (Figure 6A), which were used to solve a global 3.9-Å-resolution structure of the pentamer (Figures 6B and S8; Table S1). D1–D3 were well resolved in the pentamer map, and an atomic model could be built *de novo* only into the N-terminal part of csg, as D4 was only partially resolved, and D5 and D6 were poorly resolved (Figure S8).

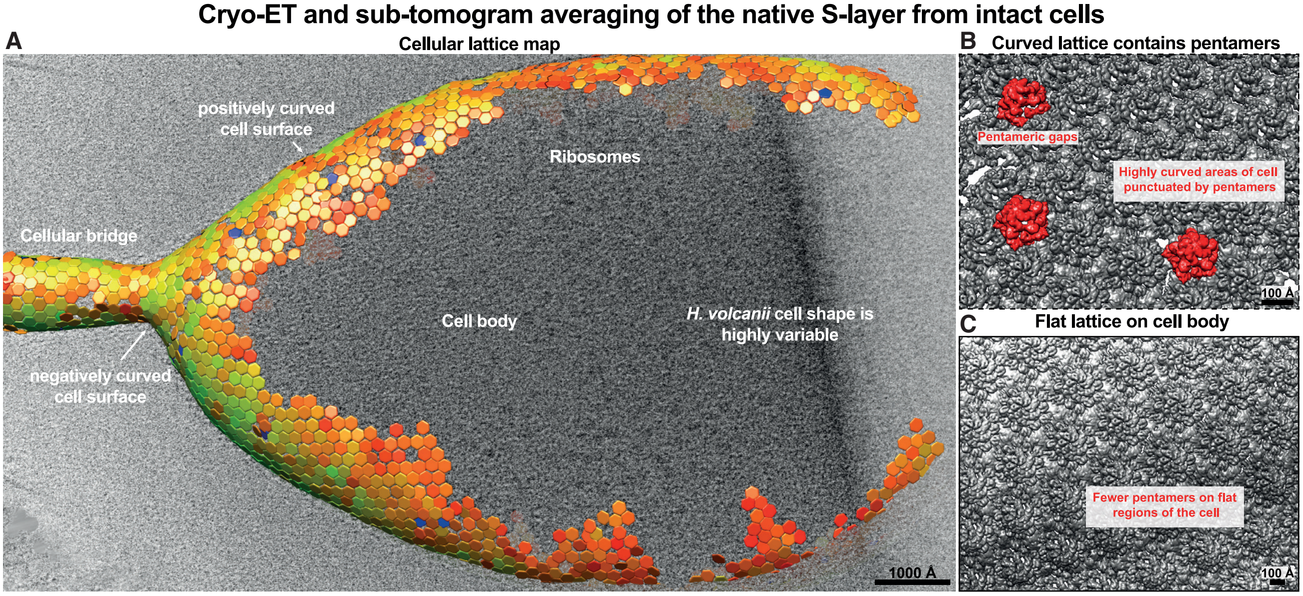

Figure 5. S-layer lattice on whole cells

(A) Cryo-ET and STA of whole cells with pleiomorphic shapes show the same hexagonal lattice with pentameric defects.

(B and C) Pentamers are increased in areas of higher membrane curvature (B), compared with flatter areas of the cell membrane (C).

The pentameric interface mediated by D1 involves the same residues as the hexamer (Figure 6C), but since the csg monomers are more tightly packed around the symmetry axis, the pore size is significantly smaller, at ~6 Å. The poor visibility of D5 and D6 suggests flexibility relative to D1–D4, possibly induced by Ho^{3+} replacement of Ca^{2+} in D5 and D6 at the lattice interfaces (Figure 6D). We infer that in the biochemical conditions employed, key interfaces within the lattice are prevented from forming, either due to a lack of Ca^{2+} or due to flexibility induced by Ho^{3+} binding, leading to isolated single particles (Figures 6E and 6F).

Complete *in situ* S-layer structure of *H. volcanii*

Cryo-EM structures of the csg pentamer and hexamer, along with our cellular tomography lattice maps, allow us to report the structure of the entire outermost surface of an archaeon (Figure 7; Video S3). The hexagonal S-layer of *H. volcanii* forms a tight and relatively impermeable sheath (Figure 7A) with a narrow pore, ~13 Å in diameter, at the hexameric axis. This S-layer harbors several key glycans pointing to the extracellular milieu (Figure 7A) and several functional metal ions stabilizing the lattice. Geometric continuity of the S-layer lattice on native membranes is maintained by pentameric defects (Figure 7B), which were also confirmed on whole *H. volcanii* cells using cryo-ET. The S-layer lattice can coat membranes with a wide range of curvatures, supporting invagination and other pleomorphic cell shapes. All these structurally observed properties of csg allow this enthralling protein to self-assemble and form a micron-scale assembly to coat archaeal cells with near-perfect continuity.

DISCUSSION

Our results will have important implications on prokaryotic cell biology and the evolution of prokaryotic and eukaryotic life. First, in the intensively studied *H. volcanii* model system, our data confirm how csg is arranged in a hexagonal S-layer tightly associated with the cell membrane, in line with previous studies using negative staining EM and cryo-EM (Kessel et al., 1988; Trachtenberg et al., 2000). On a structural level, the S-layer appears to be stabilized by several positively charged ions, most likely Ca^{2+} or Mg^{2+}, at the hexameric and trimeric interfaces, allowing co-operative assembly of the lattice and in agreement with past work (Cohen et al., 1991). Calcium dependency of S-layer assembly has been observed in previous atomic structures of Gram-negative (Bharat et al., 2017; von Kügelgen et al., 2020) and Gram-positive bacterial S-layers (Baranova et al., 2012). Further research will be needed to explore how widely this general principle of S-layer assembly is shared. However, in contrast to reported structures of bacterial S-layers (Baranova et al., 2012; Bharat et al., 2017; Fioravanti et al., 2019), there are only narrow pores seen in the csg lattice, which are further obscured by observed glycan moieties; this means that the S-layer severely restricts access to the archaeal cell membrane and the pseudo-periplasmic space, which is a known site for important cellular biochemical reactions (Albers and Meyer, 2011). Furthermore, glycans observed in our structural data have been suggested to play a vital role in the recognition of "self" during cell mating (Shalev et al., 2017), and the surface-exposed arrangement of glycan moieties agrees with that role.

Analogous to multi-domain viral capsids—such as that of HIV-1 (Mattei et al., 2016)—to close the lattice around the cell, pentamers of csg complete the S-layer (Figure 4C), and gaps as seen in bacterial S-layers (Bharat et al., 2017) were not observed. This suggests that closing gaps around the cell membrane is needed for stability, protection from harmful entities, or maintaining a pseudo-periplasmic space between the S-layer and the cell membrane (Albers and Meyer, 2011). One interesting future

CellPress
OPEN ACCESS

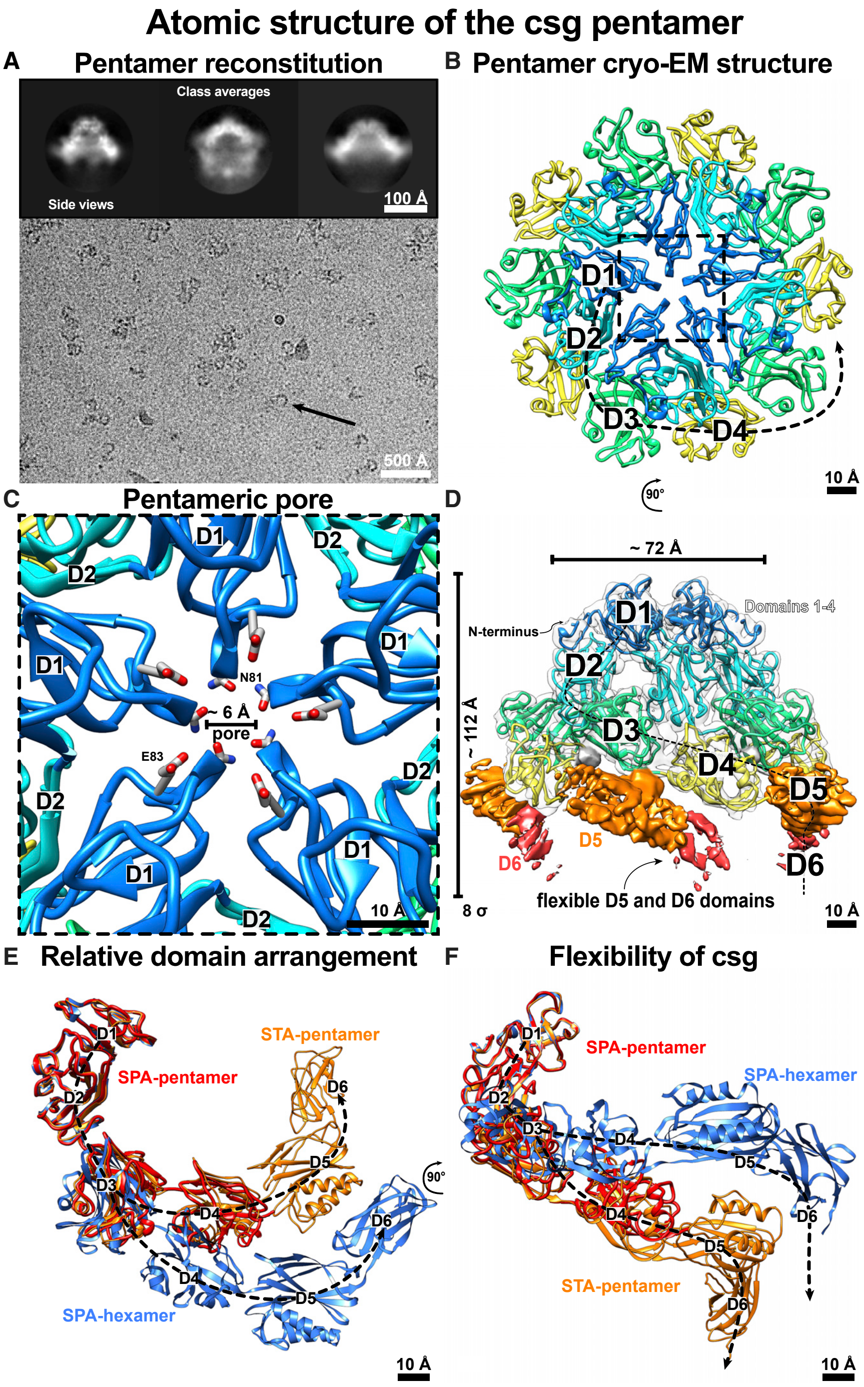

Figure 6. Atomic structure of csg pentamers

(A) Cryo-EM image of csg pentamers reconstituted with Ho^{3+} ions; single particles are marked (black arrows). Inset: class averages show side and tilted views of the pentamer.

(B) The 3.9-Å resolution structure shows csg in a pentameric structure (only resolved D1–D4 shown).

(C) The pentameric axis shows a smaller pore with D1 interactions around the axis.

(D) Only D1–D4 are resolved in the cryo-EM map, with D5 and D6 poorly resolved, indicating flexibility without the lattice binding partners (contour level lower left of panel).

(E and F) Comparison of the single-particle analysis (SPA) hexamer and pentamer structure with the pentamer model derived from STA demonstrates large changes in relative positions of the domains. D1 and D2 of all structures have been aligned.

direction of research could investigate whether the position of pentameric defects is somehow regulated by the SLP secretion machinery or whether this is a stochastic process relying on a pool of unassembled csg in the pseudo-periplasm. Another fascinating direction of future inquiry would be to understand how other cell-surface features, including flagella and pili, extend out from the cell with respect to pentameric defects in the S-layer. Our data further show how this tightly assembled S-layer can significantly deviate from a planar arrangement needed to coat cell envelopes with different curvatures during the cell cycle. Our cryo-ET experiments on vesicles and whole cells confirm that both positive (seen on vesicles and cellular membranes; Figures 4 and 5) and negative curvatures (seen in the *in vitro* tubes and polymorphic cells; Figures S6 and 5) are physiologically relevant because the cell membrane must invaginate during important processes such as mating, septation, and cell division (Abdul-Halim et al., 2020; Sivabalasarma et al., 2021).

On the technical side, solving structures from two-dimensional crystals has been notoriously difficult (Ganser-Pornillos et al., 2007; Scherer et al., 2014). In this study using STA to produce an initial reference structure, combined with single-particle cryo-EM, we solve an atomic structure from a sheet-like crystal. This will be of interest to many researchers in the cryo-EM field working on two-dimensional electron crystallography (Gonen et al., 2004; Scherer et al., 2014).

Using sequence- and structure-prediction-based bioinformatic analysis, we show conservation of the observed arrangement of Ig-like domains across several archaeal phyla, which means that this complete *in situ* structure of an archaeal S-layer will open the door to understanding how cell surfaces are organized in these microbes. Based on our bioinformatics data, we anticipate that many archaeal species, particularly haloarchaea, share these general principles of S-layer structure and organization. Nonetheless, we do anticipate there to be marked differences in S-layer anchoring and assembly mechanisms, dependent on the habitat or the organism, as suggested previously (Albers

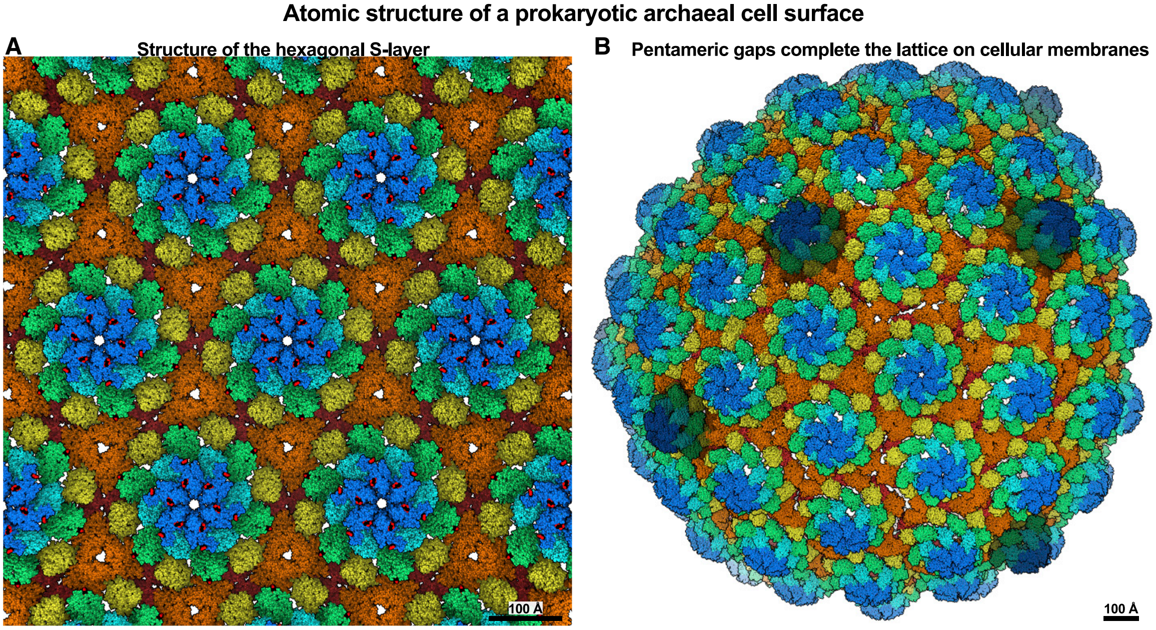

Figure 7. Atomic resolution description of an archaeal cell surface

(A) Atomic structure of the csg hexagonal S-layer lattice revealed in this study shows how a flexible but tight proteinaceous layer coats cells of the model archaeon *H. volcanii*. The coloring scheme for D1–D6 is the same as in Figure 1C.

(B) The S-layer lattice is almost perfectly continuous on cellular membranes with no gaps. The lattice is completed by pentameric defects (colored darker), allowing this fascinating array to fully encase and protect these archaea from harsh environments.

and Meyer, 2011; Bharat et al., 2021). Curiously, several haloarchaeal organisms contain multiple putative SLPs (Figure S3), suggesting that they may be able to use different SLPs to evade predators and phages or to regulate mating preferences.

We anticipate that our results will guide our understanding of certain aspects of eukaryogenesis. Primordial S-layer Ig-like domains at the cell surface may have helped in the recognition of neighboring foreign cells for the formation of mutualistic interactions (Baum and Baum, 2014), which may have led to the emergence of more sophisticated, multi-component recognition systems, such as the modern eukaryotic immune system (Baum and Baum, 2014; Imachi et al., 2020; Spang et al., 2017). Since the Asgard archaeaon "*Candidatus* Prometheoarchaeum syntrophicum" strain MK-D1—the closest known prokaryotic relative of eukaryotes to date—has been implicated to possess S-layers (Imachi et al., 2020), a fascinating question to contemplate is why and when S-layers were lost in the evolution of contemporary eukaryotes.

Beyond the fundamental biology revealed by our work, S-layers have outstanding potential for synthetic biology applications, and using our past work on S-layers (Bharat et al., 2017; von Kügelgen et al., 2020), the first applications are being reported (Charrier et al., 2019). Although this rising field is still in its infancy, there is a huge potential for the synthesis of the next generation of biomaterials, and recent prominent studies have highlighted this potential (Ben-Sasson et al., 2021). Our results, thus, further our knowledge of a captivating class of naturally abundant molecules with a marked ability to self-assemble around prokaryotic cells at high copy numbers.

Limitations of the study

At the current resolution, the unique chemical identity of the bound metal cations cannot be ascertained. Further structural and biophysical studies will be required to deduce the nature of the bound metal ions. We hope that this complete atomic structure of an archaeal cell surface will help stimulate further research into this enthralling field.

STAR★METHODS

Detailed methods are provided in the online version of this paper and include the following:

- KEY RESOURCES TABLE
- RESOURCE AVAILABILITY
  - Lead contact
  - Materials availability
  - Data and code availability
- EXPERIMENTAL MODEL AND SUBJECT DETAILS
- METHOD DETAILS
  - Purification of csg protein
  - *In vitro* reconstitution and purification of specimens for cryo-EM
  - Cryo-EM sample preparation
  - Cryo-EM and cryo-ET data collection
  - Cryo-EM single particle analysis
  - Cryo-ET data analysis
  - Model building and refinement
  - Bioinformatic analysis
- QUANTIFICATION AND STATISTICAL ANALYSIS

CellPress
OPEN ACCESS

SUPPLEMENTAL INFORMATION

Supplemental information can be found online at https://doi.org/10.1016/j.celrep.2021.110052.

ACKNOWLEDGMENTS

T.A.M.B. is a recipient of a Sir Henry Dale Fellowship, jointly funded by the Wellcome Trust and the Royal Society (202231/Z/16/Z). T.A.M.B. would like to thank the Vallee Research Foundation, the European Molecular Biology Organization, and the Leverhulme Trust for support. The authors would like to thank Thorsten Allers for the kind gift of the H26 strain of *H. volcanii*, Wim Hagen for collecting of part of the cryo-ET data in this manuscript, and Jan Löwe and Iain Duggin for helpful discussions. V.A. would like to thank Andrei Lupas for continued support. This work was partly supported by institutional funds of the Max Planck Society.

AUTHOR CONTRIBUTIONS

A.v.K. and T.A.M.B. designed research; A.v.K., V.A., and T.A.M.B. performed research; A.v.K., V.A., and T.A.M.B. analyzed data; A.v.K., V.A., and T.A.M.B. wrote the manuscript.

DECLARATION OF INTERESTS

The authors declare no competing interests.

Received: July 2, 2021
Revised: September 28, 2021
Accepted: November 1, 2021
Published: November 23, 2021

REFERENCES

Abdul Halim, M.F., Pfeiffer, F., Zou, J., Frisch, A., Haft, D., Wu, S., Tolić, N., Brewer, H., Payne, S.H., Paša-Tolić, L., and Pohlschroder, M. (2013). *Haloferax volcanii* archaeosortase is required for motility, mating, and C-terminal processing of the S-layer glycoprotein. Mol. Microbiol. *88*, 1164–1175.

Abdul-Halim, M.F., Schulze, S., DiLucido, A., Pfeiffer, F., Bisson Filho, A.W., and Pohlschroder, M. (2020). Lipid Anchoring of Archaeosortase Substrates and Midcell Growth in Haloarchaea. MBio *11*, e00349-20.

Albers, S.V., and Meyer, B.H. (2011). The archaeal cell envelope. Nat. Rev. Microbiol. *9*, 414–426.

Allers, T., Ngo, H.-P., Mevarech, M., and Lloyd, R.G. (2004). Development of additional selectable markers for the halophilic archaeon *Haloferax volcanii* based on the leuB and trpA genes. Appl. Environ. Microbiol. *70*, 943–953.

Arbing, M.A., Chan, S., Shin, A., Phan, T., Ahn, C.J., Rohlin, L., and Gunsalus, R.P. (2012). Structure of the surface layer of the methanogenic archaean *Methanosarcina acetivorans*. Proc. Natl. Acad. Sci. USA *109*, 11812–11817.

Baranova, E., Fronzes, R., Garcia-Pino, A., Van Gerven, N., Papapostolou, D., Péhau-Arnaudet, G., Pardon, E., Steyaert, J., Howorka, S., and Remaut, H. (2012). SbsB structure and lattice reconstruction unveil Ca^{2+} triggered S-layer assembly. Nature *487*, 119–122.

Baum, D.A., and Baum, B. (2014). An inside-out origin for the eukaryotic cell. BMC Biol. *12*, 76.

Ben-Sasson, A.J., Watson, J.L., Sheffler, W., Johnson, M.C., Bittleston, A., Somasundaram, L., Decarreau, J., Jiao, F., Chen, J., Mela, I., et al. (2021). Design of biologically active binary protein 2D materials. Nature *589*, 468–473.

Bepler, T., Morin, A., Rapp, M., Brasch, J., Shapiro, L., Noble, A.J., and Berger, B. (2019). Positive-unlabeled convolutional neural networks for particle picking in cryo-electron micrographs. Nat. Methods *16*, 1153–1160.

Bharat, T.A., and Scheres, S.H. (2016). Resolving macromolecular structures from electron cryo-tomography data using subtomogram averaging in RELION. Nat. Protoc. *11*, 2054–2065.

Bharat, T.A., Riches, J.D., Kolesnikova, L., Welsch, S., Krähling, V., Davey, N., Parsy, M.L., Becker, S., and Briggs, J.A. (2011). Cryo-electron tomography of Marburg virus particles and their morphogenesis within infected cells. PLoS Biol. *9*, e1001196.

Bharat, T.A.M., Russo, C.J., Löwe, J., Passmore, L.A., and Scheres, S.H.W. (2015). Advances in Single-Particle Electron Cryomicroscopy Structure Determination applied to Sub-tomogram Averaging. Structure *23*, 1743–1753.

Bharat, T.A.M., Kureisaite-Ciziene, D., Hardy, G.G., Yu, E.W., Devant, J.M., Hagen, W.J.H., Brun, Y.V., Briggs, J.A.G., and Löwe, J. (2017). Structure of the hexagonal surface layer on *Caulobacter crescentus* cells. Nat. Microbiol. *2*, 17059.

Bharat, T.A.M., von Kügelgen, A., and Alva, V. (2021). Molecular Logic of Prokaryotic Surface Layer Structures. Trends Microbiol. *29*, 405–415.

Burghardt, T., Junglas, B., Siedler, F., Wirth, R., Huber, H., and Rachel, R. (2009). The interaction of *Nanoarchaeum equitans* with *Ignicoccus hospitalis*: proteins in the contact site between two cells. Biochem. Soc. Trans. *37*, 127–132.

Burnley, T., Palmer, C.M., and Winn, M. (2017). Recent developments in the CCP-EM software suite. Acta Crystallogr. D Struct. Biol. *73*, 469–477.

Charrier, M., Li, D., Mann, V.R., Yun, L., Jani, S., Rad, B., Cohen, B.E., Ashby, P.D., Ryan, K.R., and Ajo-Franklin, C.M. (2019). Engineering the S-Layer of *Caulobacter crescentus* as a Foundation for Stable, High-Density, 2D Living Materials. ACS Synth. Biol. *8*, 181–190.

Cohen, S., Shilo, M., and Kessel, M. (1991). Nature of the salt dependence of the envelope of a Dead Sea archaebacterium, *Haloferax volcanii*. Arch. Microbiol. *156*, 198–203.

Deatherage, J.F., Taylor, K.A., and Amos, L.A. (1983). Three-dimensional arrangement of the cell wall protein of *Sulfolobus acidocaldarius*. J. Mol. Biol. *167*, 823–848.

Duggin, I.G., Aylett, C.H., Walsh, J.C., Michie, K.A., Wang, Q., Turnbull, L., Dawson, E.M., Harry, E.J., Whitchurch, C.B., Amos, L.A., and Löwe, J. (2015). CetZ tubulin-like proteins control archaeal cell shape. Nature *519*, 362–365.

Eddy, S.R. (1998). Profile hidden Markov models. Bioinformatics *14*, 755–763.

Emsley, P., Lohkamp, B., Scott, W.G., and Cowtan, K. (2010). Features and development of Coot. Acta Crystallogr. D Biol. Crystallogr. *66*, 486–501.

Fagan, R.P., and Fairweather, N.F. (2014). Biogenesis and functions of bacterial S-layers. Nat. Rev. Microbiol. *12*, 211–222.

Filloux, A., and Whitfield, C. (2016). Editorial: The many wonders of the bacterial cell surface. FEMS Microbiol. Rev. *40*, 161–163.

Fioravanti, A., Van Hauwermeiren, F., Van der Verren, S.E., Jonckheere, W., Goncalves, A., Pardon, E., Steyaert, J., De Greve, H., Lamkanfi, M., and Remaut, H. (2019). Structure of S-layer protein Sap reveals a mechanism for therapeutic intervention in anthrax. Nat. Microbiol. *4*, 1805–1814.

Förster, F., Medalia, O., Zauberman, N., Baumeister, W., and Fass, D. (2005). Retrovirus envelope protein complex structure in situ studied by cryo-electron tomography. Proc. Natl. Acad. Sci. USA *102*, 4729–4734.

Gambelli, L., Meyer, B.H., McLaren, M., Sanders, K., Quax, T.E.F., Gold, V.A.M., Albers, S.V., and Daum, B. (2019). Architecture and modular assembly of *Sulfolobus* S-layers revealed by electron cryotomography. Proc. Natl. Acad. Sci. USA *116*, 25278–25286.

Ganser-Pornillos, B.K., Cheng, A., and Yeager, M. (2007). Structure of full-length HIV-1 CA: a model for the mature capsid lattice. Cell *131*, 70–79.

Gonen, T., Sliz, P., Kistler, J., Cheng, Y., and Walz, T. (2004). Aquaporin-0 membrane junctions reveal the structure of a closed water pore. Nature *429*, 193–197.

Hagen, W.J.H., Wan, W., and Briggs, J.A.G. (2017). Implementation of a cryo-electron tomography tilt-scheme optimized for high resolution subtomogram averaging. J. Struct. Biol. *197*, 191–198.

Imachi, H., Nobu, M.K., Nakahara, N., Morono, Y., Ogawara, M., Takaki, Y., Takano, Y., Uematsu, K., Ikuta, T., Ito, M., et al. (2020). Isolation of an archaeon at the prokaryote-eukaryote interface. Nature *577*, 519–525.

Kaminski, L., Guan, Z., Yurist-Doutsch, S., and Eichler, J. (2013). Two distinct N-glycosylation pathways process the *Haloferax volcanii* S-layer glycoprotein upon changes in environmental salinity. MBio *4*, e00716, e13.

Kandiba, L., and Eichler, J. (2014). Archaeal S-layer glycoproteins: post-translational modification in the face of extremes. Front. Microbiol. *5*, 661.

Kandiba, L., Lin, C.W., Aebi, M., Eichler, J., and Guerardel, Y. (2016). Structural characterization of the N-linked pentasaccharide decorating glycoproteins of the halophilic archaeon *Haloferax volcanii*. Glycobiology *26*, 745–756.

Kerou, M., Offre, P., Valledor, L., Abby, S.S., Melcher, M., Nagler, M., Weckwerth, W., and Schleper, C. (2016). Proteomics and comparative genomics of *Nitrososphaera viennensis* reveal the core genome and adaptations of archaeal ammonia oxidizers. Proc. Natl. Acad. Sci. USA *113*, E7937–E7946.

Kessel, M., Wildhaber, I., Cohen, S., and Baumeister, W. (1988). Three-dimensional structure of the regular surface glycoprotein layer of *Halobacterium volcanii* from the Dead Sea. EMBO J. *7*, 1549–1554.

Kish, A., Miot, J., Lombard, C., Guigner, J.M., Bernard, S., Zirah, S., and Guyot, F. (2016). Preservation of Archaeal Surface Layer Structure During Mineralization. Sci. Rep. *6*, 26152.

Klingl, A. (2014). S-layer and cytoplasmic membrane - exceptions from the typical archaeal cell wall with a focus on double membranes. Front. Microbiol. *5*, 624.

Konrad, Z., and Eichler, J. (2002). Lipid modification of proteins in Archaea: attachment of a mevalonic acid-based lipid moiety to the surface-layer glycoprotein of *Haloferax volcanii* follows protein translocation. Biochem. J. *366*, 959–964.

Kremer, J.R., Mastronarde, D.N., and McIntosh, J.R. (1996). Computer visualization of three-dimensional image data using IMOD. J. Struct. Biol. *116*, 71–76.

Lechner, J., and Sumper, M. (1987). The primary structure of a procaryotic glycoprotein. Cloning and sequencing of the cell surface glycoprotein gene of halobacteria. J. Biol. Chem. *262*, 9724–9729.

Li, P.N., Herrmann, J., Tolar, B.B., Poitevin, F., Ramdasi, R., Bargar, J.R., Stahl, D.A., Jensen, G.J., Francis, C.A., Wakatsuki, S., and van den Bedem, H. (2018). Nutrient transport suggests an evolutionary basis for charged archaeal surface layer proteins. ISME J. *12*, 2389–2402.

Liebschner, D., Afonine, P.V., Baker, M.L., Bunkóczi, G., Chen, V.B., Croll, T.I., Hintze, B., Hung, L.W., Jain, S., McCoy, A.J., et al. (2019). Macromolecular structure determination using X-rays, neutrons and electrons: recent developments in Phenix. Acta Crystallogr. D Struct. Biol. *75*, 861–877.

Lu, H., Lü, Y., Ren, J., Wang, Z., Wang, Q., Luo, Y., Han, J., Xiang, H., Du, Y., and Jin, C. (2015). Identification of the S-layer glycoproteins and their covalently linked glycans in the halophilic archaeon *Haloarcula hispanica*. Glycobiology *25*, 1150–1162.

Mastronarde, D.N. (2005). Automated electron microscope tomography using robust prediction of specimen movements. J. Struct. Biol. *152*, 36–51.

Mattei, S., Glass, B., Hagen, W.J., Kräusslich, H.G., and Briggs, J.A. (2016). The structure and flexibility of conical HIV-1 capsids determined within intact virions. Science *354*, 1434–1437.

Milojevic, T., Albu, M., Blazevic, A., Gumerova, N., Konrad, L., and Cyran, N. (2019). Nanoscale Tungsten-Microbial Interface of the Metal Immobilizing Thermoacidophilic Archaeon *Metallosphaera sedula* Cultivated With Tungsten Polyoxometalate. Front. Microbiol. *10*, 1267.

Mullakhanbhai, M.F., and Larsen, H. (1975). *Halobacterium volcanii* spec. nov., a Dead Sea halobacterium with a moderate salt requirement. Arch. Microbiol. *104*, 207–214.

Murshudov, G.N., Skubák, P., Lebedev, A.A., Pannu, N.S., Steiner, R.A., Nicholls, R.A., Winn, M.D., Long, F., and Vagin, A.A. (2011). REFMAC5 for the refinement of macromolecular crystal structures. Acta Crystallogr. D Biol. Crystallogr. *67*, 355–367.

Parente, J., Casabuono, A., Ferrari, M.C., Paggi, R.A., De Castro, R.E., Couto, A.S., and Giménez, M.I. (2014). A rhomboid protease gene deletion affects a novel oligosaccharide N-linked to the S-layer glycoprotein of *Haloferax volcanii*. J. Biol. Chem. *289*, 11304–11317.

Pettersen, E.F., Goddard, T.D., Huang, C.C., Couch, G.S., Greenblatt, D.M., Meng, E.C., and Ferrin, T.E. (2004). UCSF Chimera–a visualization system for exploratory research and analysis. J. Comput. Chem. *25*, 1605–1612.

Pettersen, E.F., Goddard, T.D., Huang, C.C., Meng, E.C., Couch, G.S., Croll, T.I., Morris, J.H., and Ferrin, T.E. (2021). UCSF ChimeraX: Structure visualization for researchers, educators, and developers. Protein Sci. *30*, 70–82.

Pum, D., Toca-Herrera, J.L., and Sleytr, U.B. (2013). S-layer protein self-assembly. Int. J. Mol. Sci. *14*, 2484–2501.

Qu, K., Glass, B., Doležal, M., Schur, F.K.M., Murciano, B., Rein, A., Rumlová, M., Ruml, T., Kräusslich, H.G., and Briggs, J.A.G. (2018). Structure and architecture of immature and mature murine leukemia virus capsids. Proc. Natl. Acad. Sci. USA *115*, E11751–E11760.

Rodrigues-Oliveira, T., Souza, A.A., Kruger, R., Schuster, B., Maria de Freitas, S., and Kyaw, C.M. (2019). Environmental factors influence the *Haloferax volcanii* S-layer protein structure. PLoS ONE *14*, e0216863.

Rohou, A., and Grigorieff, N. (2015). CTFFIND4: Fast and accurate defocus estimation from electron micrographs. J. Struct. Biol. *192*, 216–221.

Rosenshine, I., Tchelet, R., and Mevarech, M. (1989). The mechanism of DNA transfer in the mating system of an archaebacterium. Science *245*, 1387–1389.

Sára, M., and Sleytr, U.B. (2000). S-Layer proteins. J. Bacteriol. *182*, 859–868.

Scherer, S., Arheit, M., Kowal, J., Zeng, X., and Stahlberg, H. (2014). Single particle 3D reconstruction for 2D crystal images of membrane proteins. J. Struct. Biol. *185*, 267–277.

Scheres, S.H. (2012). RELION: implementation of a Bayesian approach to cryo-EM structure determination. J. Struct. Biol. *180*, 519–530.

Schindelin, J., Arganda-Carreras, I., Frise, E., Kaynig, V., Longair, M., Pietzsch, T., Preibisch, S., Rueden, C., Saalfeld, S., Schmid, B., et al. (2012). Fiji: an open-source platform for biological-image analysis. Nat. Methods *9*, 676–682.

Schrödinger (2021). The PyMOL Molecular Graphics System*Version 2.0*. https://pymol.org/2/.

Shalev, Y., Turgeman-Grott, I., Tamir, A., Eichler, J., and Gophna, U. (2017). Cell Surface Glycosylation Is Required for Efficient Mating of *Haloferax volcanii*. Front. Microbiol. *8*, 1253.

Sivabalasarma, S., Wetzel, H., Nußbaum, P., van der Does, C., Beeby, M., and Albers, S.V. (2021). Analysis of Cell-Cell Bridges in *Haloferax volcanii* Using Electron Cryo-Tomography Reveal a Continuous Cytoplasm and S-Layer. Front. Microbiol. *11*, 612239.

Spang, A., Caceres, E.F., and Ettema, T.J.G. (2017). Genomic exploration of the diversity, ecology, and evolution of the archaeal domain of life. Science *357*, eaaf3883.

Steinegger, M., Meier, M., Mirdita, M., Vöhringer, H., Haunsberger, S.J., and Söding, J. (2019). HH-suite3 for fast remote homology detection and deep protein annotation. BMC Bioinformatics *20*, 473.

Sulkowski, N.I., Hardy, G.G., Brun, Y.V., and Bharat, T.A.M. (2019). A Multiprotein Complex Anchors Adhesive Holdfast at the Outer Membrane of *Caulobacter crescentus*. J. Bacteriol. *201*, e00112-19.

Sumper, M., Berg, E., Mengele, R., and Strobel, I. (1990). Primary structure and glycosylation of the S-layer protein of *Haloferax volcanii*. J. Bacteriol. *172*, 7111–7118.

Tamir, A., and Eichler, J. (2017). N-Glycosylation Is Important for Proper *Haloferax volcanii* S-Layer Stability and Function. Appl. Environ. Microbiol. *83*, e03152-16.

Tan, Y.Z., Baldwin, P.R., Davis, J.H., Williamson, J.R., Potter, C.S., Carragher, B., and Lyumkis, D. (2017). Addressing preferred specimen orientation in single-particle cryo-EM through tilting. Nat. Methods *14*, 793–796.

Taylor, K.A., Deatherage, J.F., and Amos, L.A. (1982). Structure of the S-layer of *Sulfolobus acidocaldarius*. Nature *299*, 840–842.

Trachtenberg, S., Pinnick, B., and Kessel, M. (2000). The cell surface glycoprotein layer of the extreme halophile *Halobacterium salinarum* and its relation to *Haloferax volcanii*: cryo-electron tomography of freeze-substituted cells and projection studies of negatively stained envelopes. J. Struct. Biol. *130*, 10–26.

CellPress
OPEN ACCESS

Turoňová, B., Schur, F.K.M., Wan, W., and Briggs, J.A.G. (2017). Efficient 3D-CTF correction for cryo-electron tomography using NovaCTF improves subtomogram averaging resolution to 3.4Å. J. Struct. Biol. *199*, 187–195.

von Kügelgen, A., Tang, H., Hardy, G.G., Kureisaite-Ciziene, D., Brun, Y.V., Stansfeld, P.J., Robinson, C.V., and Bharat, T.A.M. (2020). *In Situ* Structure of an Intact Lipopolysaccharide-Bound Bacterial Surface Layer. Cell *180*, 348–358.e15.

Wakai, H., Nakamura, S., Kawasaki, H., Takada, K., Mizutani, S., Aono, R., and Horikoshi, K. (1997). Cloning and sequencing of the gene encoding the cell surface glycoprotein of *Haloarcula japonica* strain TR-1. Extremophiles *1*, 29–35.

Wan, W., Kolesnikova, L., Clarke, M., Koehler, A., Noda, T., Becker, S., and Briggs, J.A.G. (2017). Structure and assembly of the Ebola virus nucleocapsid. Nature *551*, 394–397.

Yang, J., Anishchenko, I., Park, H., Peng, Z., Ovchinnikov, S., and Baker, D. (2020). Improved protein structure prediction using predicted interresidue orientations. Proc. Natl. Acad. Sci. USA *117*, 1496–1503.

Zheng, S.Q., Palovcak, E., Armache, J.P., Verba, K.A., Cheng, Y., and Agard, D.A. (2017). MotionCor2: anisotropic correction of beam-induced motion for improved cryo-electron microscopy. Nat. Methods *14*, 331–332.

Zimmermann, L., Stephens, A., Nam, S.Z., Rau, D., Kübler, J., Lozajic, M., Gabler, F., Söding, J., Lupas, A.N., and Alva, V. (2018). A Completely Reimplemented MPI Bioinformatics Toolkit with a New HHpred Server at its Core. J. Mol. Biol. *430*, 2237–2243.

Zink, I.A., Pfeifer, K., Wimmer, E., Sleytr, U.B., Schuster, B., and Schleper, C. (2019). CRISPR-mediated gene silencing reveals involvement of the archaeal S-layer in cell division and virus infection. Nat. Commun. *10*, 4797.

Zivanov, J., Nakane, T., Forsberg, B.O., Kimanius, D., Hagen, W.J., Lindahl, E., and Scheres, S.H. (2018). New tools for automated high-resolution cryo-EM structure determination in RELION-3. eLife *7*, e42166.

Zivanov, J., Nakane, T., and Scheres, S.H.W. (2020). Estimation of high-order aberrations and anisotropic magnification from cryo-EM datasets in RELION-3.1. IUCrJ *7*, 253–267.

STAR★METHODS

KEY RESOURCES TABLE

REAGENT or RESOURCE	SOURCE	IDENTIFIER
Chemicals, peptides, and recombinant proteins		
Fiducial gold (FG) 10 nm, 400 μL	CMC Utrecht	N/A
Deposited data		
Hv-csg hexameric single particle structure	This study	PDB: 7PTR
Hv-csg hexameric cryo-EM map	This study	EMDB: EMD-13634
Hv-csg pentameric single particle structure	This study	PDB: 7PTU
Hv-csg pentameric cryo-EM map	This study	EMDB: EMD-13638
Hv-csg hexameric subtomogram averaging structure	This study	PDB: 7PTT
Hv-csg hexameric cryo-ET map	This study	EMDB: EMD-13637
Hv-csg pentameric subtomogram averaging structure	This study	PDB: 7PTP
Hv-csg pentameric cryo-ET map	This study	EMDB: EMD-13632
Inverted S-layer tube cryo-ET map	This study	EMDB: EMD-13639
Experimental models: Organisms/strains		
Haloferax volcanii H-26	(Allers et al., 2004)	H-26
Software and algorithms		
3DFSC	(Tan et al., 2017)	https://3dfsc.salk.edu/
CCP-EM	(Burnley et al., 2017)	https://www.ccpem.ac.uk/
Coot	(Emsley et al., 2010)	https://www2.mrc-lmb.cam.ac.uk/personal/pemsley/coot/
CTFFIND	(Rohou and Grigorieff, 2015)	https://grigoriefflab.janelia.org/ctf
EPU	Thermo Fisher Scientific	https://www.thermofisher.com/us/en/home/electron-microscopy/products/software-em-3d-vis/epu-software.html
Fiji	(Schindelin et al., 2012)	https://fiji.sc/
IMOD	(Kremer et al., 1996)	https://bio3d.colorado.edu/imod/
HHpred	(Steinegger et al., 2019)	https://github.com/soedinglab/hh-suite
HMMER	(Eddy, 1998)	http://hmmer.org/
MATLAB R2019b	Mathworks	https://uk.mathworks.com/
MotionCor2 (implemented in RELION 3.1)	(Zheng et al., 2017)	N/A
MPI Bioinformatics Toolkit	(Zimmermann et al., 2018)	https://toolkit.tuebingen.mpg.de/
novaCTF	(Turoňová et al., 2017)	https://github.com/turonova/novaCTF
PHENIX	(Liebschner et al., 2019)	https://phenix-online.org/
PlaceObject Chimera Plugin	(Qu et al., 2018)	https://www2.mrc-lmb.cam.ac.uk/groups/briggs/resources/place-object/
PyMOL	(Schrödinger, 2021)	https://pymol.org/2/
REFMAC5	(Murshudov et al., 2011)	https://www2.mrc-lmb.cam.ac.uk/groups/murshudov/content/refmac/refmac.html
RELION 3.1	(Zivanov et al., 2020; Bharat and Scheres, 2016; Bharat et al., 2017)	https://www2.mrc-lmb.cam.ac.uk/relion
SerialEM	(Mastronarde, 2005)	https://bio3d.colorado.edu/SerialEM/
TOPAZ	(Bepler et al., 2019)	https://github.com/tbepler/topaz
TrRosetta	(Yang et al., 2020)	https://yanglab.nankai.edu.cn/trRosetta/

(*Continued on next page*)

Continued

REAGENT or RESOURCE	SOURCE	IDENTIFIER
UCSF Chimera	(Pettersen et al., 2004)	https://www.cgl.ucsf.edu/chimera/
UCSF ChimeraX	(Pettersen et al., 2021)	https://www.cgl.ucsf.edu/chimerax/
Other		
R2/2 200 mesh Cu/Rh holey carbon grids	Quantifoil	https://www.quantifoil.com/

RESOURCE AVAILABILITY

Lead contact

Further information and requests for reagents may be directed to, and will be fulfilled by the Lead Contact, Tanmay A. M. Bharat (tanmay.bharat@path.ox.ac.uk).

Materials availability

Further information and requests for reagents may be directed to, and will be fulfilled by the Lead Contact, Tanmay A. M. Bharat (tanmay.bharat@path.ox.ac.uk).

Data and code availability

- Cryo-EM maps have been deposited in the Electron Microscopy Data Bank (EMDB) under the accession code EMD-13634 for the hexameric SPA, EMD-13638 for the pentameric SPA, EMD-13637 for the hexameric STA and EMD-13632 for the pentameric STA. Corresponding refined atomic models have been deposited in the Protein Data Bank (PDB) under the accession numbers to 7PTR for the hexameric SPA, 7PTU for the pentameric SPA, 7PTT for the hexameric STA and 7PTP for the pentameric STA respectively. The cryo-ET map of the inverted S-layer tubes has been deposited with the EMDB accession code EMD-13639. For further details see Tables S1 and S2 and the Key resources table.
- This paper does not report original code.
- Any additional information required to reanalyse the data reported in this paper is available from the lead contact upon request.

EXPERIMENTAL MODEL AND SUBJECT DETAILS

All *H. volcanii* strains listed in this study are listed in the Key resources table. *H. volcanii* strains were grown in yeast-peptone-casamino acid (Hv-YPC) medium (Allers et al., 2004) at 45°C. Archaeal strains used in this study are further listed in Table S3.

METHOD DETAILS

Purification of csg protein

Wild-type, mature, cell-surface csg protein was purified from the native organism using the lectin concanavalin A. Briefly, 6 L of Hv-YPC medium (Allers et al., 2004) were inoculated 1:50 with a late-log phase culture of *H. volcanii* (strain H26, Table S3), and cells were grown aerobically at 45°C to an optical density (OD_{600}) of 1.5. Cells were harvested by centrifugation (5,000 *rcf.*, 4°C, 30 min). Cells were resuspended in 20 mL lysis buffer (20 mM HEPES/NaOH pH 7.5, 500 mM NaCl, 1 mM $MnCl_2$, 1 mM $CaCl_2$, 1x cOmplete protease inhibitor (Roche), 50 μg/mL DNaseI, 1 U/mL benzonase (SigmaAldrich)) per 1 L cell pellet and lysed by passing the suspension five times through a homogenizer at 20,000 psi. Cell debris was removed by centrifugation (35,000 *rcf.*, 4°C, 45 min), and archaeal membranes were isolated by ultracentrifugation (200,000 *rcf.*, 4°C, 3 h). Membranes were extracted by resuspending the pellet in 50 mL concanavalin A binding buffer (20 mM HEPES/NaOH pH 7.5, 500 mM NaCl, 1 mM $MnCl_2$, 1 mM $CaCl_2$, 0.04% (v/v) Triton X-100 (SigmaAldrich)) for 2 h. The protein solution was loaded onto three 5 mL HiTrap-Concanavalin-A-4B-HP columns (GE Healthcare) using an ÄKTA pure 25 system (GE Healthcare). Unbound protein was washed away with 75 mL concanavalin A binding buffer, and bound protein was eluted with an increasing gradient of 45 mL elution buffer (20 mM HEPES/NaOH pH 7.5, 500 mM NaCl, 50 mM methyl-α-D-mannopyranoside (SigmaAldrich), 0.02% (v/v) Triton X-100). Fractions containing csg were pooled, concentrated using a 30 kDa MWCO Ultra Centrifugal tube (Amicon) and loaded to a Superdex S200-Increase 10/300 GL column (GE Healthcare) equilibrated with 20 mM HEPES/NaOH pH 7.5, 150 mM $MgCl_2$, 0.65% (w/v) CHAPS detergent. Protein was eluted in the same buffer, and fractions containing csg were collected, concentrated (Amicon 30 kDa MWCO), flash-frozen in liquid nitrogen and stored at −80°C until further use. Chromatograms and SDS-PAGE gel images were visualized with MATLAB (MathWorks) and Fiji (Schindelin et al., 2012), respectively. This mature, purified csg protein was used for all experiments below, including *in vitro* reconstitution of hexagonal sheets and pentamers.

In vitro reconstitution and purification of specimens for cryo-EM

Reconstitution of S-layer tubes and sheets: S-layer tubes were assembled by adding 15 mM $CaCl_2$ to purified wild-type csg at a final protein concentration of 3.2 mg/mL and incubating the solution on ice for 25 mins. Assembled S-layer sheets were then immediately prepared for cryo-EM.

Purification of vesicles for cryo-ET and subtomogram averaging: Vesicles were purified from a 200 mL *H. volcanii* cell culture which was previously inoculated 1:50 with a late-log starter culture and grown to an optical density (OD_{600}) of 1.2 at 45°C. Cells were removed by centrifugation (5,000 *rcf*, 20 min, 25°C), and the supernatant was filtered through a 0.45 μm pore-size filter. Archaeal vesicles were isolated by ultracentrifugation (200,000 *rcf.*, 4°C, 3 h) and carefully resuspended in 10 μL of 18% (w/v) artificial seawater (SW) containing identical salt concentrations as Hv-YPC media, but lacking nutrient sources.

Reconstitution of csg pentamers: Pentamers of csg were assembled by adding 1.5-1.75 mM $HoCl_3$ to purified wild-type csg at a final protein concentration of 2.5 mg/mL and incubating the solution on ice for 2 hours.

Cryo-EM sample preparation

For cryo-EM grid preparation, 2.5 μL of *in vitro* reconstituted sample or purified vesicles solution or *H. volcanii* culture grown on a 1.5% (w/v) agar plate were applied to a freshly glow discharged Quantifoil R2/2 Cu/Rh 200 mesh grid, adsorbed for 10 s, blotted for 4-5 s and plunge-frozen into liquid ethane in a Vitrobot Mark IV (ThermoFisher), while the blotting chamber was maintained at 100% humidity at 10°C. For cryo-ET, 10 nm protein-A gold (CMC Utrecht) was additionally added to the samples immediately prior to grid preparation.

Cryo-EM and cryo-ET data collection

Cryo-EM single particle data: Single-particle cryo-EM data were collected on a Titan Krios G3 microscope (ThermoFisher) operating at 300 kV fitted with a Quantum energy filter (slit width 20 eV) and a K3 direct electron detector (Gatan) with a sampling pixel size of 0.55 Å running in counting super-resolution mode. For the csg sheets S-layer sample used for the csg hexameric lattice structure, a total of 18,468 movies were collected in two sessions with a dose rate of 3.5 e^-/pixel/s on the camera level. The sample was subjected to 3.4 s of exposure, during which a total dose of 49 or 51.44 $e^-/Å^2$ respectively was applied, and 40 frames were recorded per movie (see Table S1). For pentameric csg specimens, a total of 11,871 movies were collected in two sessions with a dose rate of 3.5 e^-/pixel/s on the camera level. The specimen was subjected to 3.6 s of exposure, during which a total dose of 53.45 or 53.9 $e^-/Å^2$ respectively was applied, and 40 frames were recorded per movie (see Table S1).

Cryo-ET data: For tomographic data collection, the SerialEM software (Mastronarde, 2005) was used as described previously (Sulkowski et al., 2019) (see Table S2). Tomographic data collection of cellular specimens was performed on the same Titan Krios microscope using the Quantum energy filter (slit width 20 eV) and the K3 direct electron detector running in counting mode. Tilt series (33 in total) with a defocus range of −5 to −8 μm were collected between ± 60° in a dose symmetric scheme (Hagen et al., 2017) with a 2° tilt increment. A total dose of 73 $e^-/Å^2$ was applied over the entire series, and image data were sampled at a pixel size of 3.468 Å. For *in-vitro* reconstituted inverted S-layer tubes 33 bi-directional and 14 dose-symmetrical) tilt series were collected with a 3° tilt increment at a dose rate of 5.88 or 6.1 e^-/pixel/s, respectively. A total dose of 90 or 123 $e^-/Å^2$ was applied over the entire series, and image data were sampled at a calibrated pixel size of 2.238 Å using a K2 direct electron detector running in counting mode (slit width 25 eV) (see Table S2). For *in situ* structure determination of the S-layer from coated vesicles, a pipeline for high-throughput data collection was adopted (Wan et al., 2017). Briefly, a Titan Krios microscope was used to collect tilt series data with a dose symmetric tilting scheme (Hagen et al., 2017). A total of 172 tilt series were collected at a pixel size of 1.328 Å, with a total dose of 120 $e^-/Å^2$ was applied over the entire series collected between ± 60° with 3° tilt increments using a K2 detector with a slit width of 20 eV.

Cryo-EM single particle analysis

Hexameric structure from two-dimensional sheets: Movies were clustered into optics groups based on the XML meta-data of the data-collection software EPU (ThermoFisher) using a k-means algorithm implemented in EPU_group_AFIS (https://github.com/DustinMorado/EPU_group_AFIS). Imported movies were motion-corrected, dose weighted, and Fourier cropped (2x) with MotionCor2 (Zheng et al., 2017) implemented in RELION3.1 (Zivanov et al., 2018). Contrast transfer functions (CTFs) of the resulting motion-corrected micrographs were estimated using CTFFIND4 (Rohou and Grigorieff, 2015). Initially, side views of S-layer sheets were first manually picked along the edge of the lattice using the helical picking tab in RELION while setting the helical rise to 160 Å. Top and tilted views were manually picked at the central hexameric axis. Manually picked particles were extracted in 4x downsampled 100 × 100 boxes and classified using reference-free 2D classification inside RELION3.1. Class averages centered at a hexameric axis were used to automatically pick particles inside RELION3.1. Automatically picked particles were extracted in 4x downsampled 100x100 pixel boxes and classified using reference-free 2D classification. Particle coordinates belonging to class averages centered at the hexameric axis were used to train TOPAZ (Bepler et al., 2019) in 5x downsampled micrographs with the neural network architecture ResNet8. For the final reconstruction, particles were picked using TOPAZ and the previously trained neural network above. Additionally, top and bottom views were picked using the reference-based autopicker inside RELION3.1, which were not readily identified by TOPAZ. Particles were extracted in 4x downsampled 100 × 100 boxes and classified using reference-free 2D classification inside RELION3.1. Particles belonging to class averages centered at the hexameric axis were combined, and particles within 100 Å were removed to prevent duplication after alignment. All resulting particles, side views from TOPAZ picking and

top/bottom views from RELION picking, were then re-extracted in 4x downsampled 100 × 100 boxes and were subjected to 3D classification using a 60 Å lowpass filtered reference map from subtomogram averaging of the inverted S-layer tubes (Figure S6). Particles from classes with the same curvature were combined, re-extracted in 400 × 400 boxes and subjected to a focused 3D auto refinement on the central 6 subunits using the scaled and lowpass filtered output from the 3D classification as a starting model. Per-particle defocus, anisotropy magnification and higher-order aberrations (Zivanov et al., 2020) were refined inside RELION3.1, followed by another round of focused 3D auto refinement and Bayesian particle polishing (Zivanov et al., 2020). At this point, both S-layer sheet datasets were merged for a final round of 3D-auto refinement after CTF Refinement. The final map was obtained from 1,087,798 particles and post-processed using a soft mask focused on the central hexamer yielding a global resolution of 3.5 Å according to the gold standard Fourier shell correlation criterion of 0.143 (Scheres, 2012). The two-dimensional sheet-like arrangement led to anisotropy in resolution, with lower resolution perpendicular to the plane as estimated by directional FSCs (Tan et al., 2017), observed previously by several studies on two-dimensional sheets (Ganser-Pornillos et al., 2007; Scherer et al., 2014). Cryo-EM single-particle data statistics are summarized in Table S1.

Structure of the csg pentamer: Images of pentameric csg complexes were similarly processed with the following differences: particles were initially picking using the Laplacian-of Gaussian algorithm implemented in RELION3.0 (Zivanov et al., 2018). Particles were extracted in 8x down-sampled in 50x50 pixel boxes and classified using reference-free 2D classification inside RELION3.0. Class averages showing high-resolution features were used for reference-based autopicking, followed by refinement as detailed above for the csg hexameric sheets. Particles belonging to 2D classes showing high-resolution features were re-extracted in 320 × 320 pixel boxes and an initial 3D reference model was prepared inside RELION3.0 with the stochastic gradient descent (SGD) algorithm. The final map (RELION3.1) was obtained from 382,105 particles and post-processed using a soft mask focused on the entire pentameric map yielding a global resolution of 3.87 Å with resolution anisotropy from 3.49-8.11 Å from the central C5 axis near domains D1-D3 (well resolved) to the more flexible domains D4 (partially resolved) and D5-D6 (not resolved).

Cryo-ET data analysis

Tilt series alignment using gold fiducials and tomogram generation was performed in IMOD (Kremer et al., 1996). Sub-tomogram averaging was performed using custom scripts written in MATLAB (MathWorks), described previously (Bharat et al., 2011; Förster et al., 2005; Wan et al., 2017). For preliminary assignment of angles and initial structure determination, we adopted previously published methods (Bharat et al., 2017) using a 3D-CTF correction method for tomographic data (Turoňová et al., 2017). This workflow allowed us to produce lattice maps from tubes, cells and vesicles of *H. volcanii* (Figures 4, 5, S6, and S7), which were used to map the positions of hexamers and pentamers in the lattice. Initial subtomogram averaging maps and output angular angle assignments were then used for sub-tomogram averaging in RELION3.1 (Bharat and Scheres, 2016; Bharat et al., 2015), leading to the final maps of the csg hexamer from tubes (15.8 Å resolution, Figure S6) and vesicles (8.0 Å resolution, Figure 4) and pentamer from vesicles (11.5 Å resolution, Figure 4). Figure panels containing cryo-EM or cryo-ET images were prepared using IMOD and Fiji (Schindelin et al., 2012). Lattice maps of S-layers were plotted inside UCSF Chimera (Pettersen et al., 2004) with the *PlaceObject* Plugin (Qu et al., 2018) and copies of atomic coordinates were plotted inside UCSF ChimeraX (Pettersen et al., 2021) with the *sym* function and the BIOMATRIX PDB file header. Supplementary videos (Videos S1, S2, and S3) were prepared with UCSF Chimera.

Model building and refinement

Csg hexameric structure from sheets: The boundaries of the six Ig-like domains, D1-D6, were predicted using HHpred (Steinegger et al., 2019) in default settings within the MPI Bioinformatics Toolkit (Zimmermann et al., 2018). Subsequently, structural models for these domains were built using the Robetta structure prediction server, employing the deep learning-based modeling method TrRosetta (Yang et al., 2020). The obtained structural models of domains D3-D6 resulted in an overall fit into the hexameric cryo-EM map of csg from the reconstituted sheets. D1-D2 deviated significantly from any obtained homology models, and for those domains, the carbon backbone of the csg protein was manually traced through a single subunit of the hexameric cryo-EM density using Coot (Emsley et al., 2010). Due to the edge effect of the box used in the refinement of the 3.5 Å map, parts of D6 displayed edge artifacts. These artifacts were removed using single-particle cryo-EM refinement in a larger box, which led to an overall slightly lower resolution (3.8 Å) but allowed fitting of the D6 homology model unambiguously. Following initial manual building (for D1-D2) or fitting in of structural models (for D3-D6), side chains were assigned in regions with density corresponding to characteristic aromatic residues allowing us to deduce the register of the amino acid sequence in the map. Another important check of the model building was the position of known glycan positions, which were readily assigned based on large unexplained densities on characteristic asparagine residues. The atomic model was then placed into the hexameric map in six copies and subjected to several rounds of refinement using refmac5 (Murshudov et al., 2011) inside the CCP-EM software suite (Burnley et al., 2017) and PHENIX (Liebschner et al., 2019), followed by manually rebuilding in Coot (Emsley et al., 2010). Model validation was performed in PHENIX and CCP-EM, and data visualization was performed in Chimera, ChimeraX, and PyMOL (Schrödinger, 2021). To analyze lattice interfaces, multiple copies of the hexameric structure were placed in the cryo-EM map prepared with a larger box size and refined once in PHENIX.

Csg pentamer: The initial manual build of D1-D2 was performed independently using the csg pentameric cryo-EM map, which served as an additional validation of the manual building performed in the csg hexamer above. The manual building exercise yielded a nearly identical result to the hexamer; thus, the final refined hexameric structures of D1-D2, along with D3-D4 were taken and fitted into the pentameric map (~3.87 Å resolution in D1-D3, lower in D4 which is partially resolved). Five copies of these D1-D4 were used

for refinement and model building as for the hexamer, except D3 and D4 was restrained in position, due to steadily deteriorating resolution in this part of the map. D5-D6 were not resolved in the pentameric structure and were thus not included in the refinements. Model validation and visualization were performed in the same way as for the hexameric structure.

Bioinformatic analysis

All sequence similarity searches were performed in the MPI Bioinformatics Toolkit (Zimmermann et al., 2018) using the sensitive sequence comparison methods in HMMER (Eddy, 1998) and HHpred (Steinegger et al., 2019). HMMER searches were performed against the nr_arc database, a version of the NCBI nonredundant protein sequence database (nr) filtered for archaeal sequences, to identify homologs of csg in haloarchaea and other closely related classes of archaea. The searches were seeded with the protein sequence of csg (UniProt ID P25062) as well as the experimentally characterized SLPs of *Halobacterium salinarum* (B0R8E4), *Haloarcula japonica* (Q9C4B4), and *Haloarcula hispanica* (csg1- G0HV85, csg2 - G0HV86). Divergent homologs of csg were detected in *Haloferax mediterranei* (two homologs), and outside haloarchaea, in the order Methanomicrobiales (e.g., *Methanospirillum hungatei*). The domain organizations of several obtained matches were analyzed using HHpred searches over the PDB70 and ECOD70 databases in default settings and using the TrRosetta method implemented within the Robetta structure prediction server (Yang et al., 2020). Additionally, the domain organization of some other experimentally characterized SLPs were also analyzed: *Aeropyrum pernix* (Q9YEG7), *Metallosphaera sedula* (SlaA - A4YHQ8, SlaB - A4YHQ9), *Methanocaldococcus jannaschii* (Q58232), *Methanosarcina acetivorans* (Q8TSG7), *Methanothermus fervidus* (P27373), *Nanoarchaeum equitans* (Burghardt et al., 2009) (Q74MU7), *Nitrososphaera viennensis* (Kerou et al., 2016) (SlaA - A0A060HS03, SlaB - A0A060HR06), and *Staphylothermus marinus* (Q54436). The SLPs of *Methanosarcina acetivorans*, *Methanocaldococcus jannaschii*, and *Nanoarchaeum equitans* contain no Ig-like domains and instead contain multiple copies of a different β sandwich fold (Arbing et al., 2012); while the former contains a membrane anchor preceded by a conserved PGF motif, the latter two are predicted to contain a small C-terminal Rossmann-like domain.

QUANTIFICATION AND STATISTICAL ANALYSIS

See Method details and Tables S1 and S2 for further information on the statistical analyses for bioinformatics and resolution estimates for cryo-EM maps.

Cell Reports, Volume *37*

Supplemental information

Complete atomic structure of a native archaeal cell surface

Andriko von Kügelgen, Vikram Alva, and Tanmay A.M. Bharat

Supplemental information

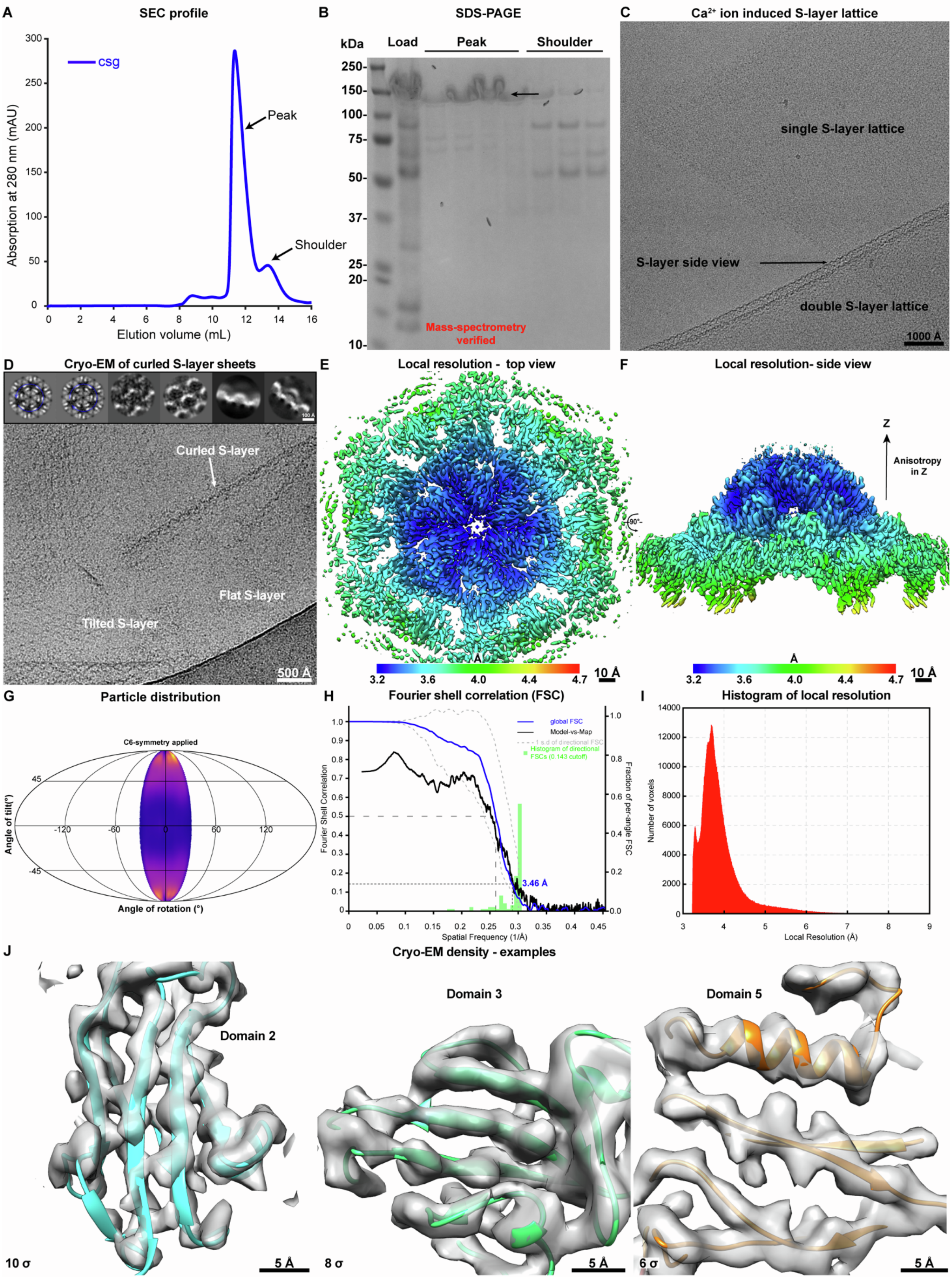

Figure S1. Cryo-EM structure of the csg from two-dimensional sheets (related to Figure 1)

(A) Size-exclusion chromatography (SEC) profile and (B) corresponding SDS-PAGE analysis of purification of csg protein. (C) Incubation of the purified protein with Ca^{2+} led to the formation of two-dimensional sheets (density black), which (D) had variable lattice curvatures. Inset – top, bottom, tilted and side views could be seen in class averages. (E-F) Local resolution estimated in RELION, plotted into the map, shown in two orthogonal orientations. (G) Angular distribution of the particles in the data set show, (H) directional resolution anisotropy, also seen in other two-dimensional crystals. 3D Fourier shell correlation (FSC) as determined by (Tan et al., 2017). Histogram binning size is set to 20. (I) Histogram of local resolutions in voxels of the cryo-EM map. (J) Examples of the csg model built into the reconstructed cryo-EM map of domains D2, D3, and D5 (contour level at the lower left).

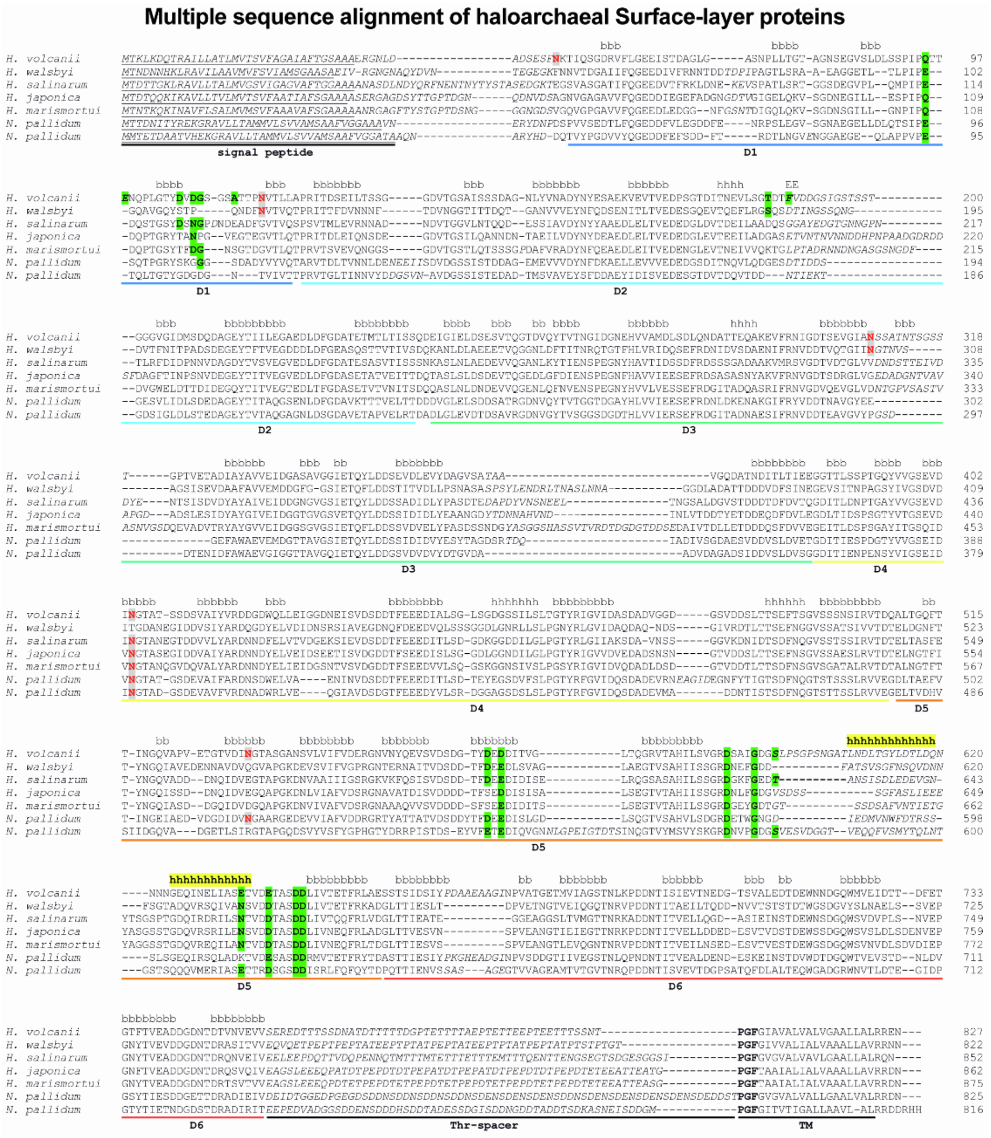

Figure S2. Multiple sequence alignment of haloarchaeal SLPs (related to Figure 2)

Multiple sequence alignment of haloarchaeal SLPs against the csg sequence. An initial alignment was calculated with MSAProbs (Liu et al., 2010) in default settings, and it was subsequently curated manually using pairwise alignments generated with HHpred (pairwise mode in default settings). Residues that could not be aligned are italicized. Domains D1-D6 are marked (underlined with the same color) as in Figure 1B, along with secondary structure observed in our cryo-EM structure (h=α-helix, b=β-strand). D1 is the most divergent in sequence, possibly due to its exposed position and potential role in surface interactions (Sumper et al., 1990). Known glycosylation sites of csg are shown in bold red with grey

highlighting, while conserved cation-binding residues in bold black with green highlighting. A lipid modification is carried out by the archaeosortase A enzyme at the C-terminus after it recognizes and cleaves the tripartite segment, comprising a conserved Pro-Gly-Phe (PGF) motif, a transmembrane helix, and a cluster of basic residues, found at the C-terminus of csg (Abdul Halim et al., 2013). This PGF motif at the C-terminus of the protein after the threonine spacer, important for S-layer anchoring to the membrane, is additionally highlighted (bold black). The experimentally characterized signal peptide of *H. volcanii* and the signal peptides predicted using SignalP5.0 (Almagro Armenteros et al., 2019) for the other SLPs are highlighted. The α-hairpin in D5 that forms a prominent trimeric interface is shown in black bold with yellow highlighting. The NCBI accession IDs of the sequences in the alignment are provided in the legend of Figure S3.

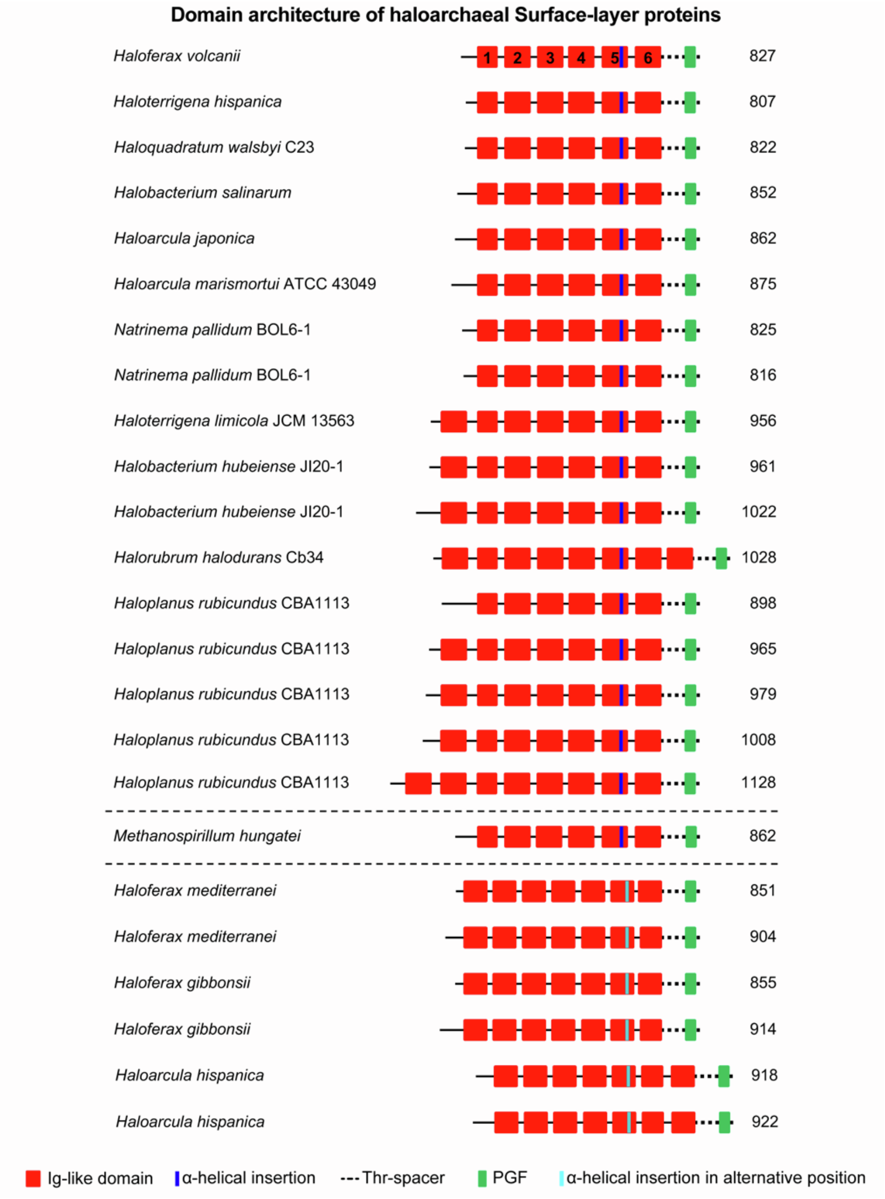

Figure S3. Domain organization of haloarchaeal SLPs (related to Figure 2)

Schematic domain diagrams of haloarchaeal SLPs are shown with the Ig-like domains shown as red boxes. The α-hairpin motif seen in D5 of csg is conserved and found in exactly one domain of other SLPs, usually the penultimate domain before the membrane anchor. This suggests that the cation bound α-helical insertion could be structurally important across haloarchaea, shown here to form the critical trimeric interface in the S-layer lattice. In more divergent haloarchaeal SLPs, such as the ones from *Haloferax mediterranei* and *Haloarcula hispanica*, this helical insertion is found in a different location, usually also in the penultimate domain before the membrane anchor. The NCBI accession IDs of the shown proteins are:

Haloferax volcanii (WP_013035656.1), *Haloterrigena hispanica* (WP_092934807.1), *Haloquadratum walsbyi* C23 (WP_014555115.1), *Halobacterium salinarum* (WP_012289536.1), *Haloarcula japonica* (WP_049909326.1), *Haloarcula marismortui* ATCC 43049 (WP_049938478.1), *Natrinema pallidum* BOL6-1 (WP_006184325.1, ELY78918.1), *Haloterrigena limicola* JCM 13563 (WP_008013949.1), *Halobacterium hubeiense* JI20-1 (CQH46446.1, WP_082687139.1), *Halorubrum halodurans* Cb34 (WP_094534449.1), *Haloplanus rubicundus* CBA1113 (WP_157969218.1, AXG06270.1, WP_114584258.1, AXG07870.1 – start codon was corrected, WP_114587121.1), *Methanospirillum hungatei* (WP_011449226.1, is not a haloarchaeon but contains putative homologous SLP), *Haloferax mediterranei* (WP_004057339.1, WP_004059902.1), *Haloferax gibbonsii* (WP_049904939.1, WP_049905079.1), and *Haloarcula hispanica* (WP_014040752.1, WP_014040753.1). Some haloarchaeal organisms, e.g., *H. rubicundus*, *H. hispanica*, and *H. mediterranei*, contain multiple putative SLPs.

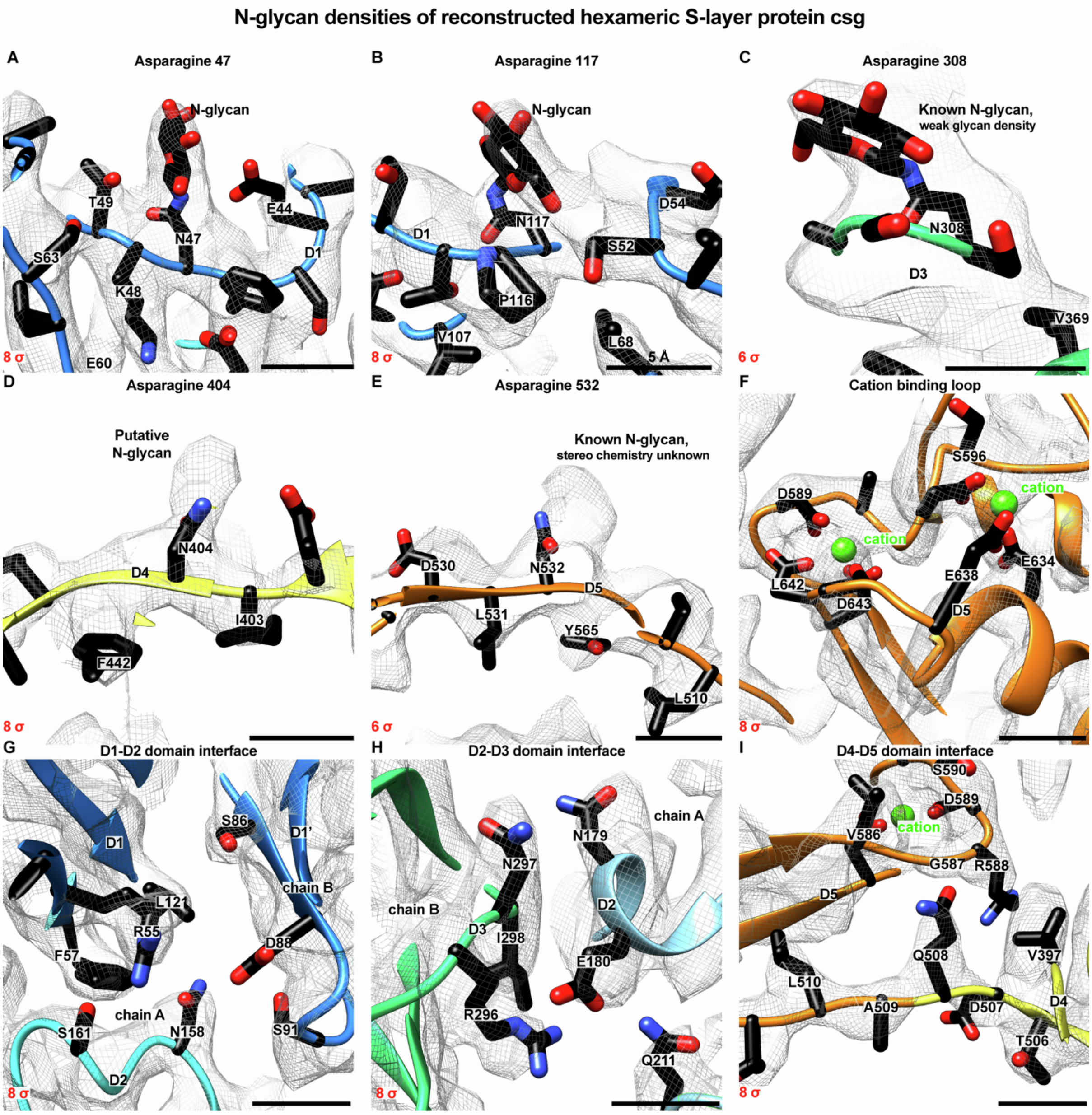

Figure S4. N-linked glycans and cation-binding sites in the csg structure (related to Figures 1 and 3)

(A-E) N-linked glycans resolved in the cryo-EM structure. For N47, N117 and N308 the first sugar has been built to illustrate the glycan location. N532 is a known glycan site, with an undefined stereochemical structure, and was thus not built into the density. Additionally, a large unexplained density was observed at N404, which we predict as hitherto undescribed glycan site (no model built). Only glycan sites that could be resolved and assigned unambiguously are discussed in this study. (F-I) Gallery of still images of the atomic model

shown inside the csg density with F), cation-binding site made of conserved amino acid residues (see Figure S2) and (G-I), domain interfaces.

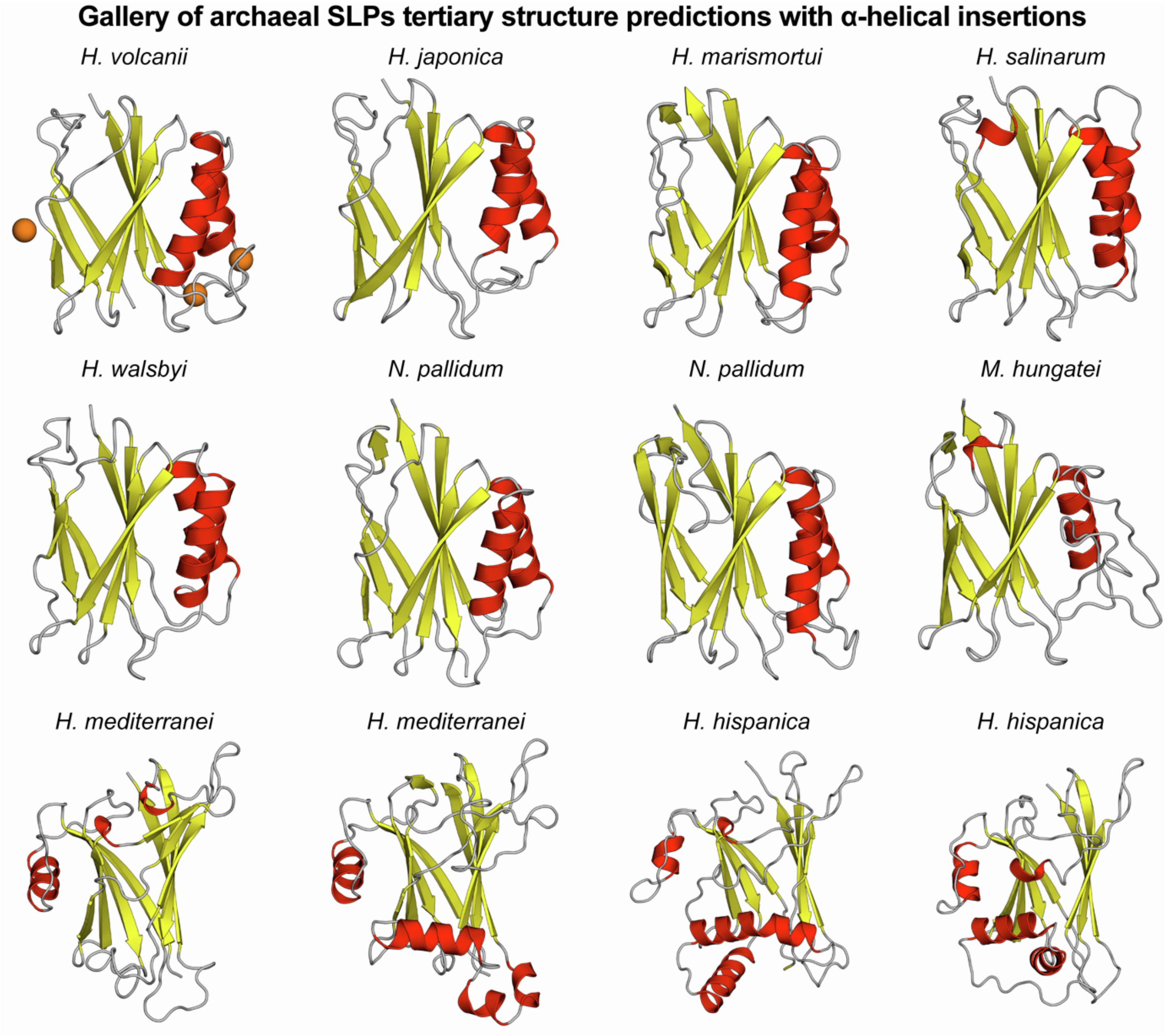

Figure S5. Gallery of SLP domains containing α-helical insertions (related to Figures 1, 2 and 3)

The penultimate domain before the membrane anchor in csg and most haloarchaeal SLPs contains an α-hairpin. In the *H. volcanii* S-layer, this hairpin forms a prominent trimeric interface and homologous regions in other haloarchaeal SLPs are expected to form a similar interface in other S-layers too. Structural models of this domain from several haloarchaeal SLPs were built using the TrRosetta method implemented within the Robetta structure prediction server. In the shown models, α-helices are colored red and β-strands yellow; in the structure of *H. volcanii* D5, bound cations are colored orange. In SLPs that exhibit low pairwise sequence similarities to csg, such as SLPs of *Haloferax mediterranei* and *Haloarcula hispanica*, the α-hairpin occurs in a different location, typically also in the penultimate domain

before the membrane anchor. Outside Haloarchaea, putative SLPs in archaea of the order Methanomicrobiales (e.g., *Methanospirillum hungatei*) also contain such α-helical insertions. The NCBI accession IDs of the proteins are provided in the legend of Figure S3.

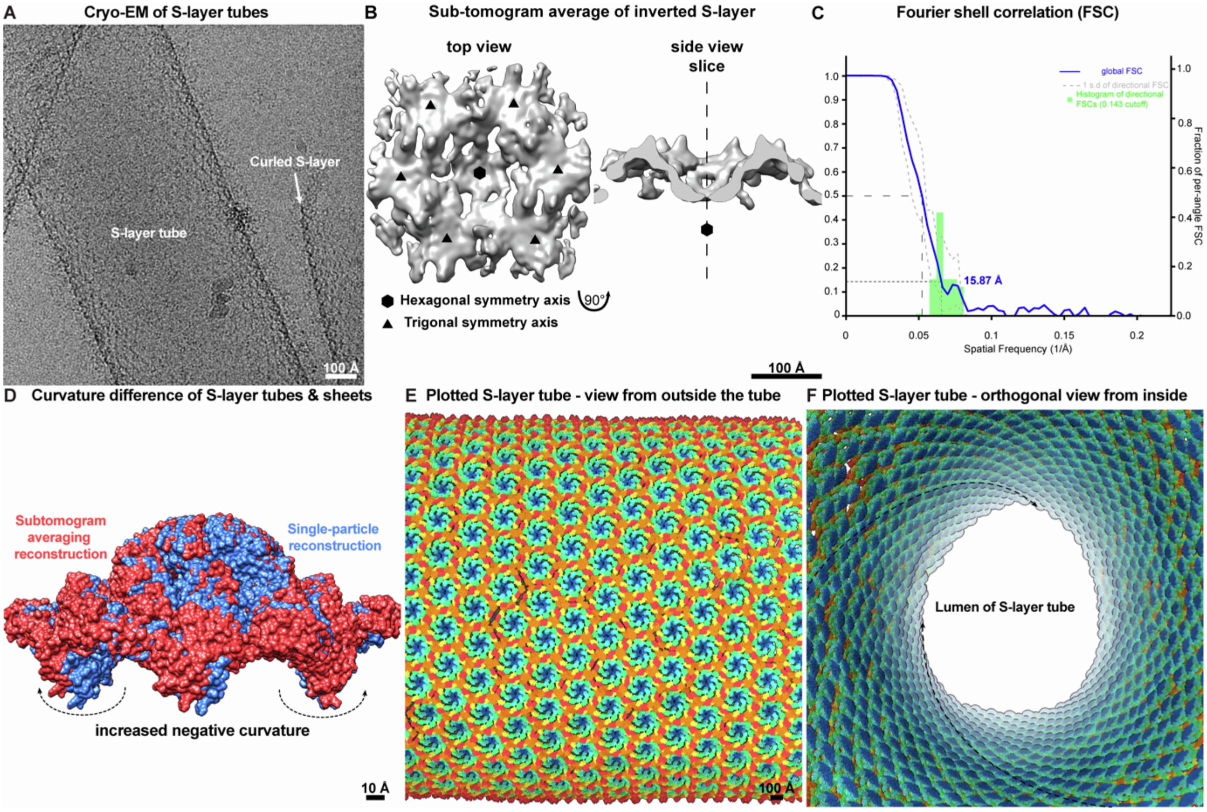

Figure S6. Subtomogram averaging structure of *in vitro* tubes of csg (related to Figure 3)

(A) Cryo-EM image of an *in vitro* S-layer tube. (B) Subtomogram averaging shows an inverted S-layer with a hexameric arrangement. (C) 3D Fourier shell correlation (FSC) with directional resolution anisotropy as described in (Tan et al., 2017). Histogram binning size is set to 7. (D) Comparison of the 15.8 Å resolution subtomogram averaging structure from tubes with the single-particle cryo-EM structure of sheets shows deviation in the D5-D6 region. (E) The subtomogram averaging map plotted onto the surface of the tube, view from the outside, E and inside the tube, F.

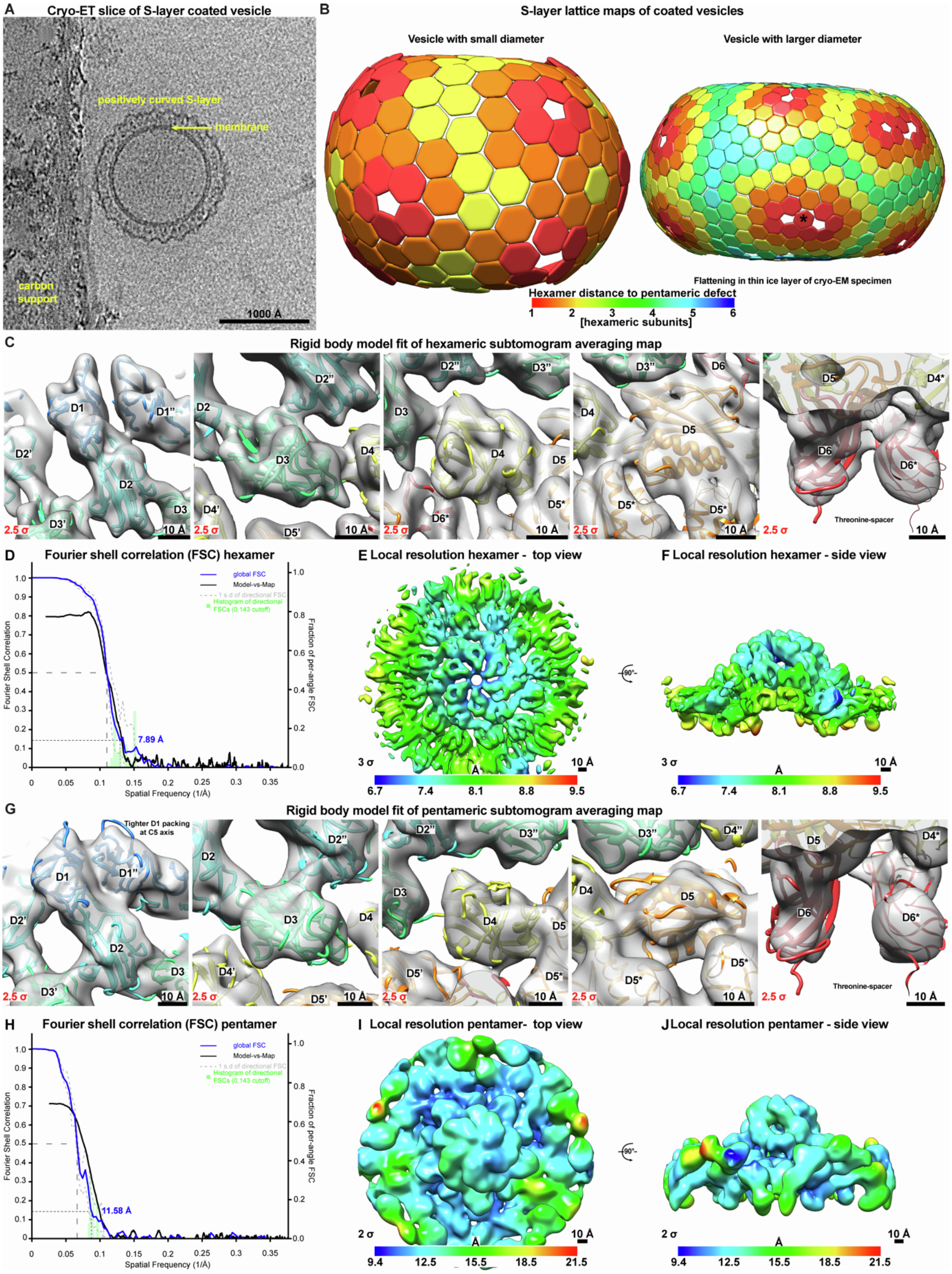

Figure S7. Subtomogram averaging structures of the csg hexamers and pentamers on native membranes (related to Figure 4)

(A) Cryo-ET slice of an isolated S-layer coated membrane vesicle of *H. volcanii* used for subtomogram averaging. (B) Lattice maps (Qu et al., 2018) of the three-dimensional coordinates and orientations of hexameric S-layer positions are colored in a red to blue scale depending on their distance to pentameric defects. A potential heptagon adjacent to pentameric defects is denoted with * (Deatherage et al., 1983). (C) Rigid body model fit of all six domains of the csg model into the hexameric subtomogram averaging map (contour level lower left panel, ‘, ‘’ and * denote domains from other csg monomers). (D) 3D Fourier shell correlation (FSC) of the hexameric subtomogram averaging map as determined by (Tan et al., 2017). Histogram binning size is set to 10. (E-F) Local resolution estimated in RELION, plotted into the hexameric subtomogram averaging reconstruction (contour level lower left panel). (G) Rigid body model fit of all five domains of the csg model into the pentameric subtomogram averaging map. (H) 3D Fourier shell correlation (FSC) of the pentameric subtomogram averaging map as determined by (Tan et al., 2017). Histogram binning size is set to 10. (I-J) Local resolution estimated in RELION, plotted into the pentameric subtomogram averaging reconstruction (contour level lower left panel).

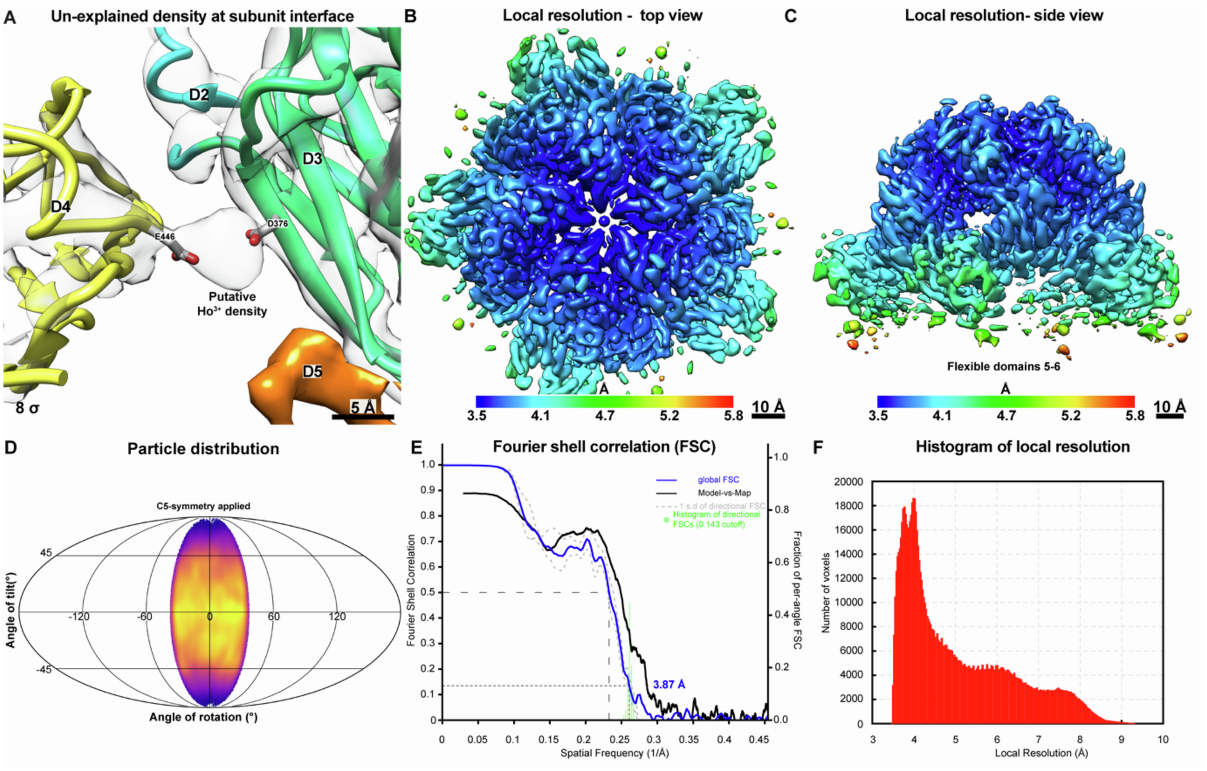

Figure S8. Cryo-EM single particle structure of the csg *in vitro* reconstituted pentamer (related to Figure 6)

(A) Unexplained strong density in the pentamer cryo-EM map, not observed in the hexamer map, possibly from Ho^{3+} ions, since no Ca^{2+} was used for the preparation of this specimen. (B-C) Local resolution estimated in RELION, plotted onto the map. (D) Angular distribution of the particles in the data set. (E) 3D Fourier shell correlation (FSC) as determined by (Tan et al., 2017). Histogram binning size is set to 16. (F) Histogram of local resolutions in voxels of the cryo-EM map.

Table S1. Cryo-EM data collection, refinement and validation statistics. (related to Figures 1 & 6)

	#csg hexamer (EMD-13634) (PDB 7PTR)		#csg pentamer (EMD-13638) (PDB 7PTU)
**Data collection and processing**			
Microscope	Titan Krios G3	Titan Krios G3	Titan Krios G3
Magnification	81,000	81,000	81,000
Voltage (kV)	300	300	300
Electron exposure (e^{-}/Å^{2})	49	51.44	53.45
Slit width (eV)	20	20	20
Detector	K3 (Gatan)	K3 (Gatan)	K3 (Gatan)
Defocus range (μm)	-1 to -3	-1 to -3	-1 to -3
Acquisition Mode	Super-resolution	Super-resolution	Super-resolution
Pixel size (Å)	0.55	0.55	0.55
AFIS[&] Mode	Yes	Yes	Yes
Micrographs collected	6,437	12,031	11,871
Micrographs used	5,875	10,906	11,357
**Data processing**			
Software reconstruction	RELION3.1(Zivanov et al., 2020)	RELION3.1(Zivanov et al., 2020)	RELION3.1(Zivanov et al., 2020)
Software picking	TOPAZ(Bepler et al., 2019)/RELION3.1	TOPAZ(Bepler et al., 2019)/RELION3.1	RELION3.0
Initial particle images (no.)	3,747,186	6,811,183	1,773,652
Final particle images (no.)	1,087,798		382,105
Rescaled Box-size Class2D (px)	100 x 100		50 x 50
Rescaled Box-size Class3D (px)	100 x 100 x 100		320 x 320 x 320
Final Box-size (px)	400 x 400 x 400		320 x 320 x 320
Pixel size final reconstruction (Å)	1.1		1.1
Symmetry imposed	C6		C5
Map resolution (Å)	3.46		3.87
FSC threshold	0.143		0.143
Map resolution range (Å)	3.23 - 4.74		3.49 - 8.11
Map sharpening *B* factor (Å^{2})	-143.26		-170.68
3DFSC sphericity[#]	0.925		0.815
**Model Refinement**			
Initial model used (PDB code)	None		None
Software	PHENIX(Liebschner et al., 2019)		PHENIX(Liebschner et al., 2019)
Model resolution (Å)	3.8		4.0
FSC threshold	0.5		0.5
Model composition			

Non-hydrogen atoms	31,710	17,035
Protein residues	4,350	4,350
Ligand residues	18	5
Ions	18	0
B factors (Å^2)		
Protein	128.24	211.75
Ligand	106.37	153.19
R.m.s. deviations		
Bond lengths (Å)	0.002	0.003
Bond angles (°)	0.454	0.787
Validation		
MolProbity score	1.55	1.80
Clashscore	6.91	8.94
Poor rotamers (%)	0.00	0.26
Cβ outliers (%)	0.00	0.00
CABLAM outliers (%)	1.94	3.62
EMRinger Score	1.24	1.51
Ramachandran plot		
Favored (%)	97.03	95.34
Allowed (%)	2.97	4.66
Disallowed (%)	0.00	0.00

[&] AFIS: Aberration Free Imaging Shift Mode.

[#] 3D-FSC sphericity as determined by the methods described in (Tan et al., 2017).

Table S2. Cryo-ET data collection, refinement and validation statistics. (Related to Figures 3 & S6)

	#csg S-layer tube inverted (EMD-13639)		# csg vesicle hexamer (EMD-13637) (PDB 7PTT)	# csg vesicle pentamer (EMD-13632) (PDB 7PTP)
**Data collection and processing**				
Microscope	Titan Krios G3	Titan Krios G3	Titan Krios	
Magnification	64,000	64,000	105,000	
Voltage (kV)	300	300	300	
Total Electron exposure ($e^-/Å^2$)	90	123	120	
Slit width (eV)	20	25	20	
Detector	K2 Summit	K2 Summit	K2 Summit	
Defocus range (µm)	-2 to -5	-2.5 to -3.5	-1.5 to -3.75	
Pixel size (Å)	2.238	2.238	1.327	
Tilt-series increment	±3°	±3°	±3°	
Tilt-series scheme	bi-directional	dose-symmetric[&]	dose-symmetric[&]	
Tilt-series range	±60°	±60°	±60°	
Tilt-series collected	33	14	172	
Tilt-series used	12	12	127	
**Data processing**				
Software tilt-series alignment	IMOD(Kremer et al., 1996)		IMOD(Kremer et al., 1996)	IMOD(Kremer et al., 1996)
Software final reconstruction	RELION3.1(Zivanov et al., 2020)		RELION3.1(Zivanov et al., 2020)	RELION3.1(Zivanov et al., 2020)
Initial particle images (no.)	25,843		83,713	3,495
Final particle images (no.)	25,843		53,063	1,640
Rescaled Box-size Class3D (px)	128 x 128 x 128		96 x 96 x 96	192 x 192 x 192
Final Box-size (px)	128 x 128 x 128		192 x 192 x 192	192 x 192 x 192
Pixel size final reconstruction (Å)	2.238		1.327	1.327
Symmetry imposed	C2		C6	C5
Map resolution (Å)	15.87		7.968	11.58
FSC threshold	0.143		0.143	0.143
Map resolution range (Å)	13 - 24		6.7-9.5	9.4-21.5
Map sharpening *B* factor ($Å^2$)	*none*		*none*	*none*
3DFSC sphericity[#]	0.953		0.969	0.985
**Model Refinement**				
Initial model used (PDB code)	N.D.		7PTR	7PTR

Software	-	PHENIX(Liebschner et al., 2019)	PHENIX(Liebschner et al., 2019)
Model resolution (Å)	N.D.	9.1	13.1
FSC threshold	N.D.	0.5	0.5
Model composition			
Non-hydrogen atoms	N.D.	31488	26240
Protein residues	N.D.	4350	2625
R.m.s. deviations			
Bond lengths (Å)	N.D.	0.003	0.003
Bond angles (°)	N.D.	0.616	0.608
Validation			
MolProbity score	N.D.	1.99	2.00
Clashscore	N.D.	14.77	15.06
Poor rotamers (%)	N.D.	0.00	0.00
Cβ outliers (%)	N.D.	0.00	0.00
CABLAM outliers (%)	N.D.	3.47	3.47
Ramachandran plot			
Favored (%)	N.D.	95.41	95.41
Allowed (%)	N.D.	4.59	4.59
Disallowed (%)	N.D.	0.00	0.00

[&] Dose-symmetrical tilt-scheme as described in (Hagen et al., 2017).

[#] 3DFSC sphericity as determined by the methods described in (Tan et al., 2017).

N.D. not determined

Table S3: Archaeal strains used in this study (related to STAR methods).

Strain or plasmid	Description or construction	Source or reference
**Strains**		
Haloferax volcanii		
H-26	*H. volcanii* Δ*pyrE2*	(Allers et al., 2004)
